# Synthesis of 2-Oxazolines from *N*-Allyl and *N*-Propargyl Amides

**DOI:** 10.3390/molecules30224369

**Published:** 2025-11-12

**Authors:** Karolina Bojar, Danuta Branowska, Ewa Wolińska

**Affiliations:** Faculty of Science, University of Siedlce, 3 Maja 54 Str., 08-110 Siedlce, Poland; karolina.bojar@uws.edu.pl (K.B.); danuta.branowska@uws.edu.pl (D.B.)

**Keywords:** 2-oxazolines, *N*-allyl amides, *N*-propargyl amides, electrophilic intramolecular cyclization

## Abstract

2-Oxazolines are five-membered heterocyclic compounds with significant biological properties. They also play an important role in organic synthesis, acting as chiral ligands and protecting groups for hydroxyamino acids and amino alcohols. Poly(2-oxazolines) are known coating materials, for example, in biomedicine. Classic synthetic methods of 2-oxazolines involve a dehydrative cyclisation reaction between amino alcohols and carboxylic acids, acid chlorides, nitriles, imidates, and aldehydes. However, the electrophilic intramolecular cyclization of unsaturated amides is becoming an increasingly important synthetic method for the preparation of 2-oxazolines. This brief review summarizes procedures for synthesizing oxazolines using the electrophilic intramolecular oxidative cyclisation of *N*-allyl and *N*-propargyl amides, as published between 2014 and 2024. It covers the synthesis of 5-halomethyl-, 5-trifluoromethyl-, 5-sulfonylmethyl-, 5-sulfenylmethyl-, 5-selenylmethyl-, 5-acetoxymethyl-, 5-hydroxymethyl-, 5-aminomethyl-, 5-alkilo-, and 5-alkylideneoxazolines.

## 1. Introduction

**Figure 1:**
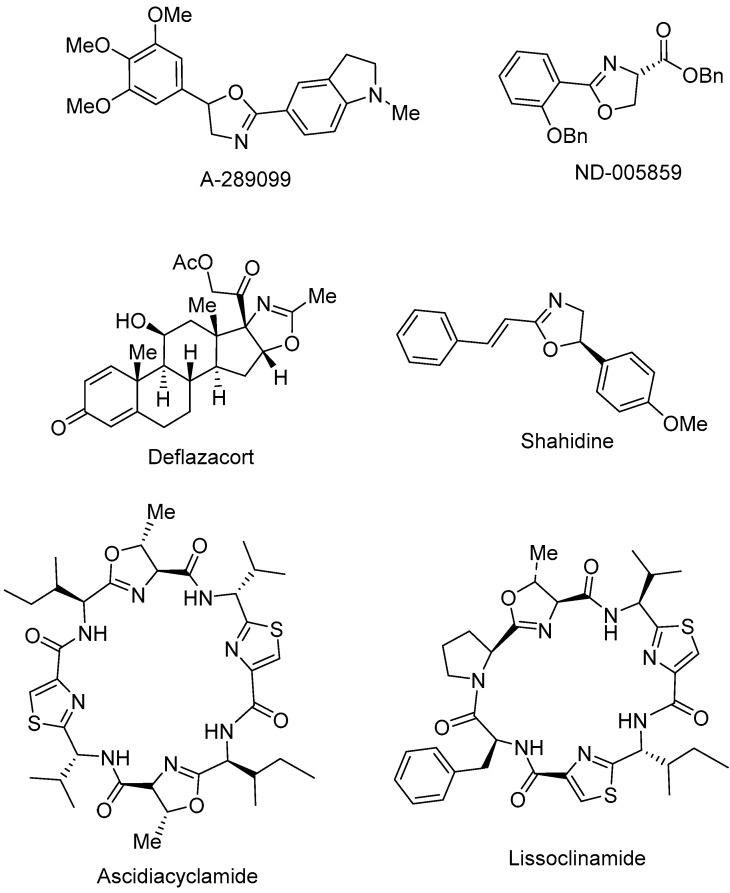
Some biologically active oxazoline derivatives.

2-Oxazoline is a prevalent structural unit that can be found in natural products, pharmaceuticals, and biologically active molecules (Figure 1) [1]. For example, oxazoline ND-005859 is an antituberculosis agent and a component of other more complex compounds with similar biological activity [2]. Deflazacort is a synthetic glucocorticosteroid drug with anti-inflammatory and immunosuppressive effects. The derivative A-289099 has been recognized as a tubulin polymerisation inhibitor, making it a potent and orally active antimitotic agent against various cancer cell lines [3]. Antibacterial Shahidine, a compound isolated from *Aegle marmelos*, is one of the naturally occurring oxazoline derivatives [4]. The oxazoline ring in Shahidine can easily undergo hydrolytic opening, producing aegeline, which has antidiabetic activity [4,5]. This finding suggests that oxazolines may be precursors of many naturally occurring compounds. A large number of biologically active compounds containing 2-oxazoline has been isolated from marine organisms, particularly sponges and ascidians. For example, ascidiacyclamide and lissoclinamide, which are cyclic, oxazoline-containing peptides, were isolated from the tunicate *Lissoclinum patella* and have been found to possess cytotoxic and antineoplastic activities [6,7].

**Figure 2:**
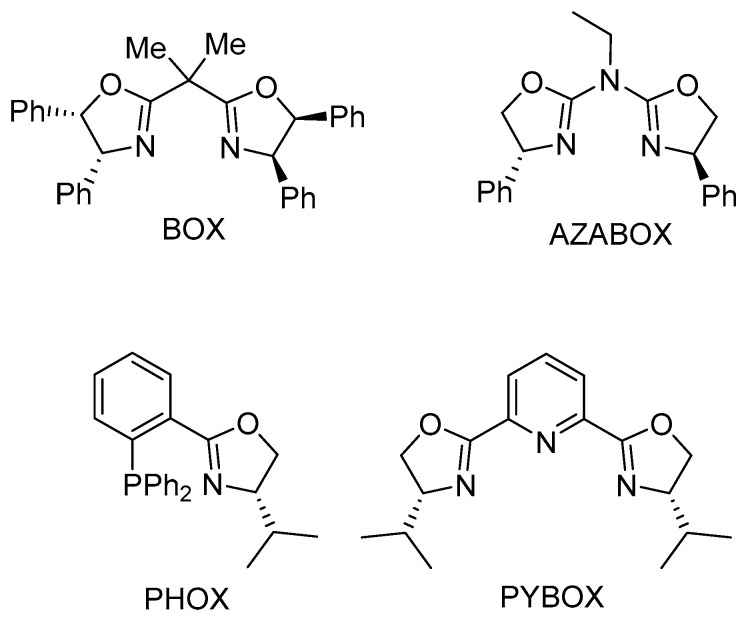
Some chiral oxazoline ligands.

In addition to their great biological significance, oxazolines are versatile building blocks in organic synthesis [8]. The ability of the oxazoline ring to open easily is a key feature that makes it a suitable protecting group for hydroxyamino acids and amino alcohols. Electrophilic-initiated cationic polymerisation of oxazolines results in poly(2-oxazoline)s, a biocompatible polymer with a wide range of applications, for example, as a coating material or in biomedicine [9,10]. However, the greatest importance of chiral oxazoline derivatives in organic synthesis stems from their successful use as ligands in asymmetric catalysis. Chiral oxazoline ligands were first used in asymmetric catalysis in 1986 by Bruner and Pfaltz. The former described a pyridine–oxazoline ligand in the asymmetric monophenylation of diols [11]. Pfaltz, on the other hand, used his semicorrin oxazoline ligand in his asymmetric cyclopropanation of olefins [12]. Since then, research interest in chiral oxazoline ligands has increased, and a vast number of structurally different oxazoline ligands has been obtained. Many structures of oxazoline ligands combine oxazoline rings and different heterocyclic systems, such as pyridine [13,14], triazine [15,16,17,18,19,20,21], pyridazine [22], quinazoline [23], and others [24,25,26]. The most active oxazoline ligands belong to the group of Box, PyBox, PHox, Pyox, and others (Figure 2). It has been proven that oxazoline ligands can catalyze various types of asymmetric transformations such as cyclopropanation and the oxidation of olefins, reduction in aromatic ketones, and carbon–carbon bond-forming reactions such as the Friedel–Crafts alkylation of indoles with different reagents, Diels–Alder reactions, Heck reactions, allylic alkylation, nitroaldol reactions, and many others, and, therefore, they have been called privileged ligands. The application of oxazoline ligands in asymmetric catalysis has been the subject of numerous reviews [27,28,29].

**Figure 3:**
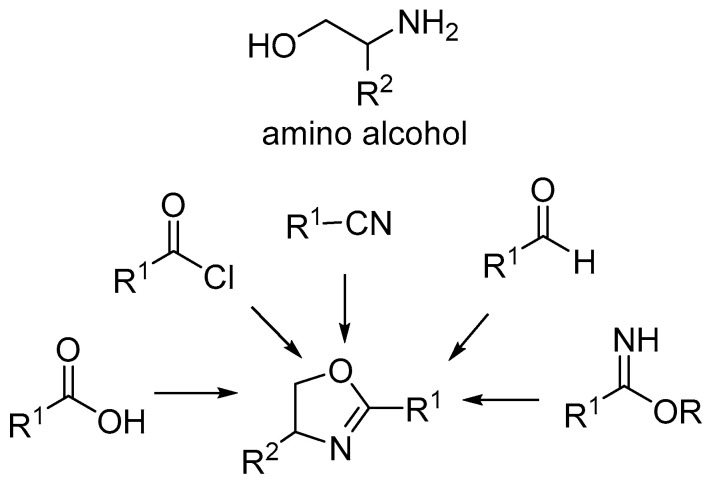
Classical substrates for oxazoline synthesis from amino alcohols.

Because of their great significance, many synthetic routes toward oxazoline derivatives have been investigated and developed by researchers around the world. Basic starting materials for obtaining chiral oxazoline derivatives are relatively inexpensive enantiomerically pure amino alcohols derived from natural amino acids. These can be used to synthesize the five-membered oxazoline ring in a variety of ways. In these classical processes, amino alcohol undergoes a reaction with carboxylic acids or their derivatives, acid chlorides and nitriles, aldehydes, and imidates (Figure 3). Although these reactions are common, they have limitations. Direct one-step condensation of carboxylic acids with amino alcohols requires highly acidic conditions and high temperatures [30]. Synthesis from acid chlorides usually proceeds in a good yield, but it is a three-step synthesis in which *N*-(β-hydroxyethyl)amides are first obtained. Then the hydroxy group is converted to a good leaving group in a reaction with mesyl chloride or with SOCl_2_. In the third stage, basic dehydrative cyclization to the oxazoline ring occurs. Therefore, such drastic reaction conditions do not appear to be appropriate for the highly functionalised biologically active derivatives. A variety of reagents active under milder conditions, including DAST [31,32], XtalFluor [33], PPE (polyphosphoric acid esters) [34], Ph_3_P/DDQ [35], Ph_3_P/DEAD [36], and Burgess reagent [37], have been proven to efficiently promote the dehydrative cyclization of acids and *N*-(β-hydroxyethyl)amides to the oxazoline ring. However, because these cofactors are used in stoichiometric quantities, large amounts of by-products are produced alongside the oxazoline, causing the purification problems. Microwave technology was also adopted to support the cyclization of acids and *N*-(β-hydroxyethyl)amides to the oxazoline ring [34,38]. Imidates react easily with alcohols to give oxazoline derivatives in good yields, but the limitation of this synthesis method is due to the need to obtain the appropriate imidate. Despite this, the method continues to attract interest. Recently, several imidate derivatives have been successfully subjected to one-pot cyclization reactions with dimethyl (±)-phosphoserinate, resulting in the formation of 2-alkyl and 2-aryl 4-phosphorylated oxazolines [39]. Cyclization of amino alcohols with aldehydes gives intermediate oxazolidine, which is then oxidized to oxazoline using NBS or another oxidizing agent [40,41,42]. The condensation of amino alcohols with nitriles, catalyzed by a Lewis acid and accompanied by the elimination of ammonia, usually requires a long time and high temperature to be completed. In many cases, the application of microwave-assisted methodology can overcome these difficulties [34,43,44].

**Scheme 1:**
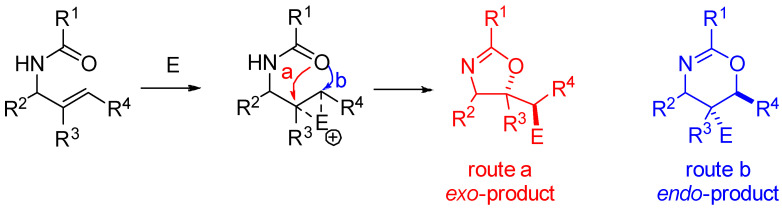
Two modes of intramolecular cyclization of *N*-allyl amides.

Recently, the electrophilic intramolecular cyclization of unsaturated amides has been recognized as an attractive and powerful tool for the synthesis of oxazoline derivatives due to the readily available substrates and relatively mild reaction conditions. An electrophile plays the role of an activator of the double or triple carbon–carbon bond towards subsequent attack of the oxygen atom of the amide carbonyl group to complete the intramolecular cyclization. When *N*-allyl amides are subjected to cyclization, the oxygen atom can attack at either electrophilic carbon atom, leading to the *exo*-product (oxazoline) and/or *endo*-product (Scheme 1). The regioselectivity of cyclization depends on the amide structure and the reaction conditions. If the *N*-allyl amide has an R^3^ substituent at the double bond, it undergoes *exo*-cyclization to oxazoline derivatives, and oxazine derivatives are not formed. On the other hand, if the amide is substituted with an R^4^ substituent, the corresponding dihydrooxazines are produced. Cyclization occurs at the atom where better stabilization is favoured by the presence of the substituent. Depending on the reaction conditions, R^4^-substituted amides may behave differently. For instance, under basic conditions, they may undergo exo-cyclization; under acidic conditions, they may undergo endo-cyclization [45]. Oxazolines obtained by the cyclization of *N*-allyl amides have a stereocenter at the C5 position of the ring. Therefore, stereoselective cyclization is a powerful strategy for constructing enantiomerically enriched heterocycles prevalent in pharmaceuticals and ligands.

**Scheme 2:**
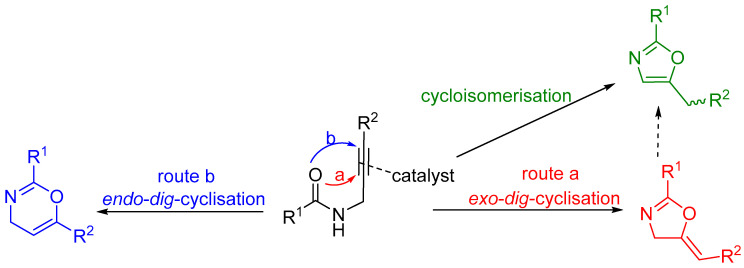
Modes of intramolecular cyclization of *N*-propargylic amides.

*N*-Propargyl amides can undergo intramolecular cyclization towards three different heterocyclic compounds (Scheme 2). Simple *exo-dig* cyclization facilitates 5-alkylideneoxazoline derivatives, while *endo-dig* cyclization leads to a six-membered oxazine. In turn, oxazole derivatives are formed in a process called cycloisomerization. Oxazoline is an intermediate in the latter transformation that is undergoing isomerization, but it usually cannot be isolated.

This review summarizes reports on the synthesis of 2-oxazolines from *N*-allyl and *N*-propargyl amides published between 2014 and 2024. Works published in 2025 thus far have also been included. Synthetic strategies have been divided into two main sections according to the starting amides: *N*-allyl amides and *N*-propargyl amides. The section on the synthesis from *N*-allyl amides is subdivided into chapters based on the functional group introduced into the oxazoline structure.

## 2. Synthesis of Oxazolines from *N*-Allyl Amides

### 2.1. Halofunctionalized Oxazolines

**Scheme 3:**
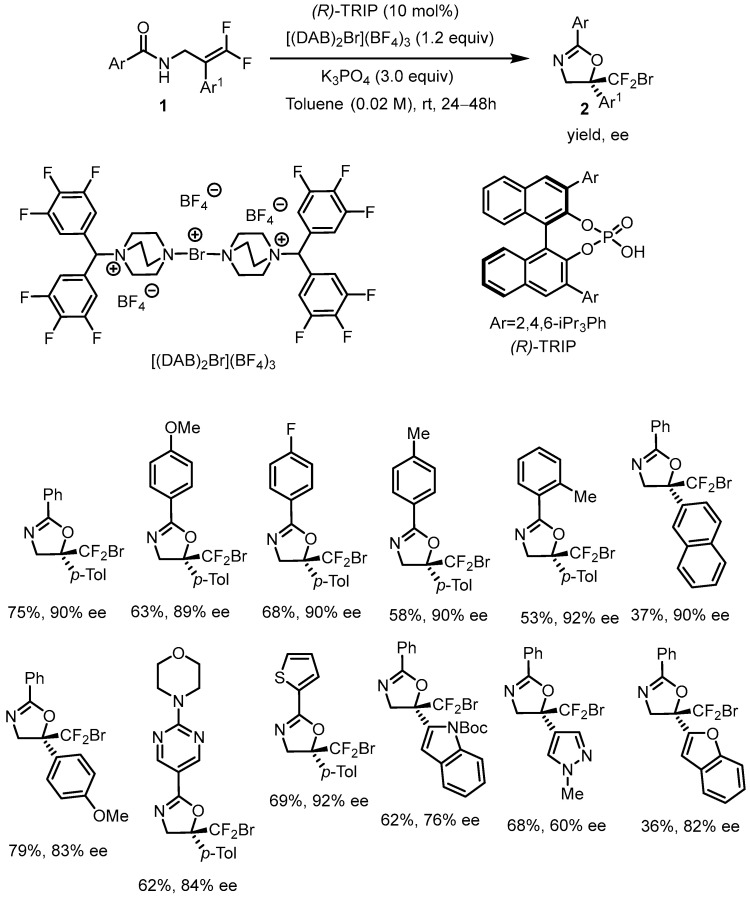
Toste bromocyclization of difluoroallylic amides using DABCOnium-based brominating reagent.

**Scheme 4:**
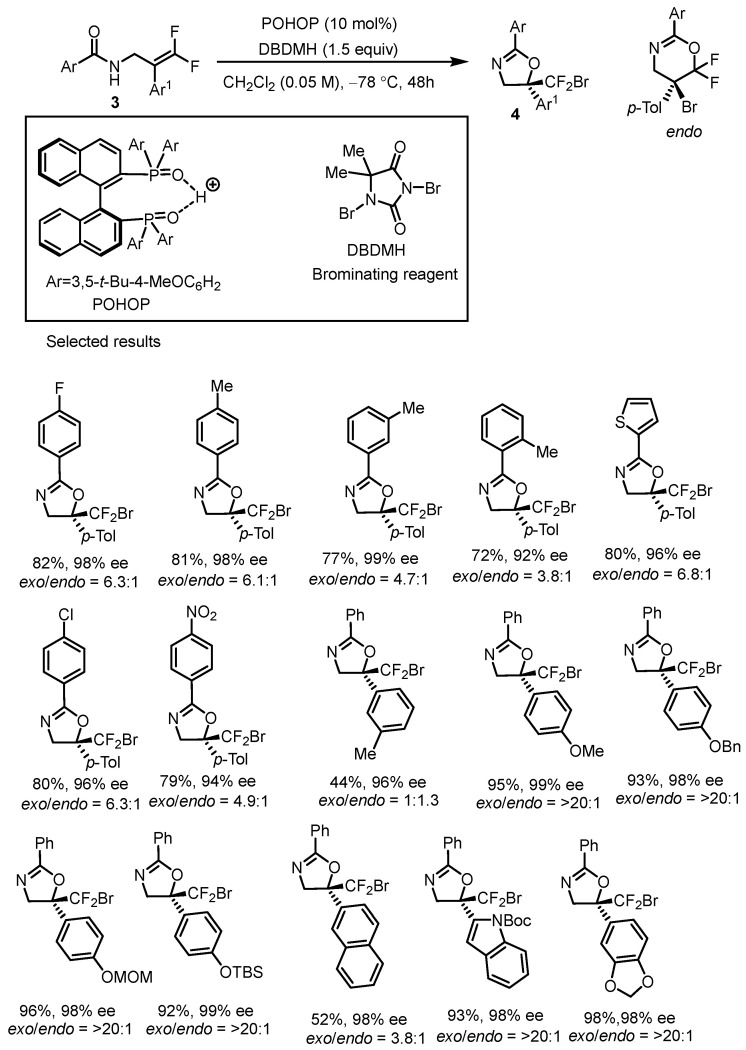
Hirokawa bromocyclization of difluoroallylic amides using DBDMH as brominating reagent.

In 2020, Toste and coworkers reported the reaction of regio- and enantioselective bromocyclization of difluoroallylic amides **1** by employing a chiral anion phase-transfer (CAPT) catalyst [46]. They have developed an approach for synthesizing chiral oxazolines **2** with tetrasubstituted stereocenters bearing the bromodifluoromethyl group, which is a privileged structural motif in the field of medicinal chemistry (Scheme 3). Several DABCOnium-based brominating reagents [(DAB)_2_Br](BF_4_)_3_ were tested as sources of electrophilic bromine in the presence of 10 mol % (*R*)-TRIP acting as the phase-transfer catalyst. The cyclization leads to a mixture of bromodifluoromethyloxazoline and *endo*-cyclization product, a 1,3-oxazine derivative. A variety of DABCOnium-based brominating reagents modified in the phenyl ring with electron-donating and electron-accepting groups were tested to assess the impact of the halogen source on the regio- and enantioselectivity of the cyclization products. The brominating reagent [(DAB)_2_Br](BF_4_)_3_, containing two perfluoro phenyl groups, yielded the bromodifluoromethyloxazoline as the main regioisomer with a yield of up to 79% and enantioselectivity of up to 90%. The steric and electronic nature of the nucleophilic difluoroallylic amide did not have any significant impact on the reaction yield and enantioselectivity. Modifying the aryl ring of the amide by introducing electron-donating groups or *para*-fluorine had little effect on the yield and enantioselectivity. Also well tolerated were heteroaromatic rings such as thiophene and pyrimidine. A more significant effect on the reaction yield and enantioselectivity was observed by changing the substituent Ar^1^ at the carbon–carbon double bond. The introduction of naphthalene as Ar^1^ provided the product with a yield of 37% and high enantioselectivity of 90% ee. The presence of a heterocycle ring at this position caused a slight reduction in enantioselectivity (*N*-Boc-indole—62% yield, 76% ee; pyrazole—68% yield, 60% ee; and benzofurane—36% yield, 82% ee). Although Toste’s reaction is a good way to access oxazolines with a bromodifluoromethylated stereocenter, a limitation of this strategy is the poor accessibility of the DABCOnium reagents, which must be synthesized in a time-consuming multistep synthesis. To overcome this limitation, Hirokawa modified the Toste method by changing the catalyst to chiral proton-bridged bisphosphine oxide complex (POHOP) and the brominating reagents to the more common *N*-bromosuccinimide (NBS), *N*-bromophthalimide (NBP), dibromoisocyanuric acid (DBI), and 1,3-dibromo-5,5-dimethylhydantoin (DBDMH) (Scheme 4) [47]. The latter appeared to be the most effective brominating reagent. The reactions at −60 °C enabled the production of chiral oxazolines **4** with yields ranging from 44 to 99% and enantioselectivity ranging from 92 to 99%. The yield and enantioselectivity of oxazoline formation are higher than under Toste conditions, but the regioselectivity is lower. In only a few cases, the observed ratio of the *exo*- and *endo*-products exceeds 20:1. The practical utility of the protocol was demonstrated by performing a gram-scale experiment in which appropriate optically active oxazoline was obtained in high yield without any loss of regioselectivity or enantioselectivity. Furthermore, the chiral catalyst was fully recovered after the general work-up. The chiral bisphosphine oxide complex, POHOP, reacts with a brominating reagent to generate a molecular bromine and an equimolar amount of BINAP dioxide. The latter serves as a bifunctional catalyst, with one phosphine oxide acting as a Lewis base to provide an active chiral P=O^+^–Br species via halogen bonding interaction and the other phosphine oxide as a Brønsted base to activate the amide substrate.

Toste and Horikawa elaborated on the bromocyclization of *N*-difluoroallyl amides **1** and **3** substituted in styrenal position. The advantage of the Hirokawa method is that it uses a widely known and commercially available brominating agent (Table 1). On the contrary, the Toste DABconium brominating agent requires time-consuming multistep synthesis. The Hirokawa catalytic system produces products with better yield and enantioselectivity (Table 1). The type of aromatic substituent (Ar^1^) in the styrenal component of the *N*-difluoroallyl amide has a crucial effect on the yield and enantioselectivity in both methods. The *N*-difluoroallyl amides used as substrates in both methods must be synthesized in a two-step process, which is undoubtedly a major limitation of both methods.

Toste and Horikawa presented enantioselective methods of synthesis of oxazolines that possess a tetrasubstituted C(sp3)-CF_2_Br moiety. The importance of such compounds arises from the fact that the CF_2_Br group can be easily transferred to a diverse set of enantioenriched difluoromethylene-containing compounds by substitution of the bromine. The difluoromethylene unit (CF_2_R/CF_2_H) can impart a variety of unique pharmacological properties to molecules of pharmacological importance such as increased lipophilicity, oxidative stability, and modulated bioavailability. Additionally, the CF_2_H group can act as both a lipophilic mimic of polar functional groups and a bioisostere of alcohols and thiols.

**Scheme 5:**
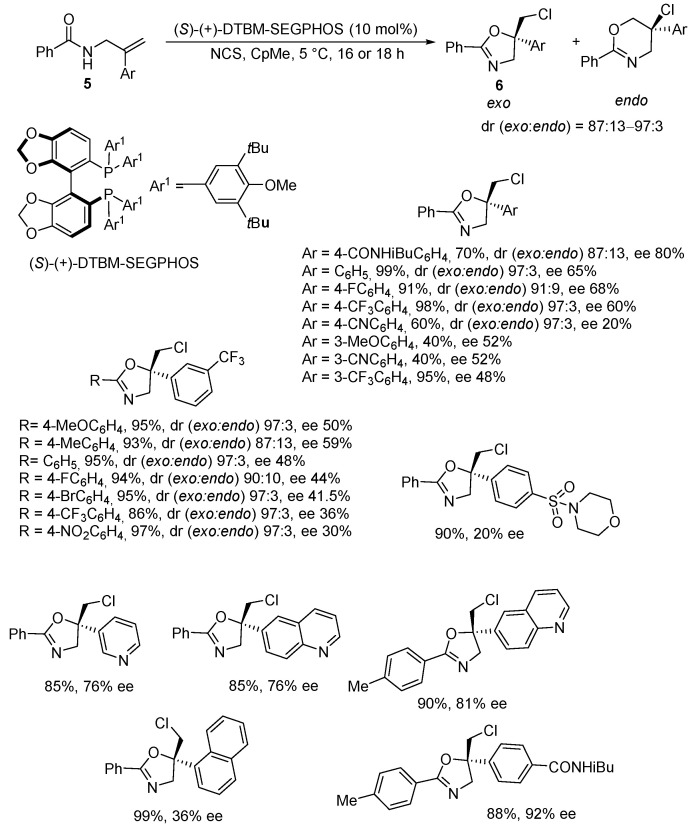
Rassias chlorocyclization of *N*-allyl amides using *N*-chlorosuccinimide (NCS) as the chlorinating reagent.

Methods of asymmetric organocatalyzed halocyclisation of *N*-allyl amides were also elaborated. The methodology usually uses chiral phosphine compounds as the organocatalyst. Enantioselective chlorocyclization of *N*-allyl amides **5** using (*S*)-(+)-DTBM-SEGPHOS as the chiral organocatalyst and *N*-chlorosuccinimide (NCS) as the chlorinating reagent provided enantioenriched 5-chloromethyloxazolines **6** in good yield, excellent diastereoselectivity, and moderate to good enantioselectivity (Scheme 5) [48]. Nevertheless, this reaction is significant because it enables products of satisfactory purity to be obtained without chromatography. This methodology, described by Rassias, is also attributed with excellent regioselectivity.

**Scheme 6:**
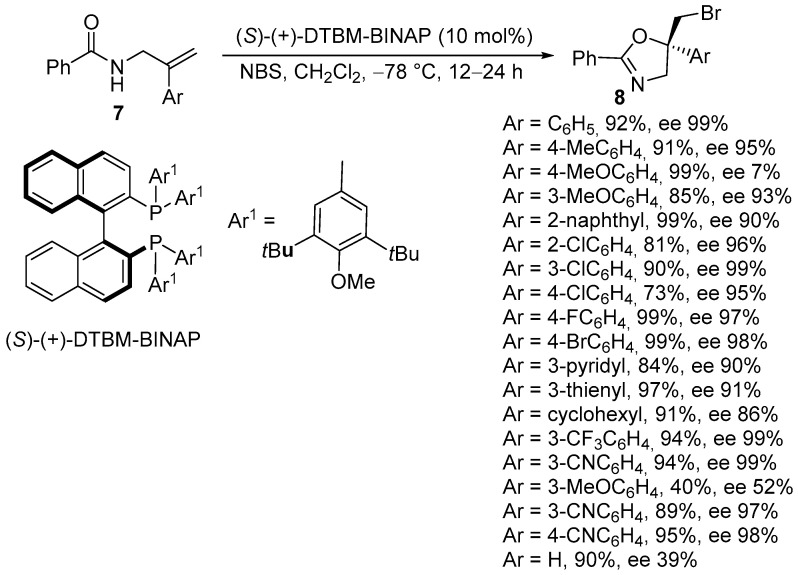
Hamashima bromocyclization of difluoroallylic amides using NBS as brominating reagent.

A notably improved enantiocontroling catalytic system for the bromocyclization of *N*-allyl amides **7** was elaborated by Hamashima (Scheme 6) [49]. Application of (*S*)-DTBM-BINAP as a Lewis base chiral catalyst and *N*-bromosuccinimide (NBS) as the bromination agent allowed us to obtain bromomethylated oxazolines **8** with excellent yields and enantioselectivities. The proposed catalytic mechanism of the catalysis involves generation in situ DTBM-BINAP monoxide as the enantioselective catalytic species in this reaction.

To compare Rassias and Hamashima’s methods of asymmetric organocatalyzed halocyclization of *N*-allyl amides, it is necessary to note that extreme cryogenic temperatures were required to achieve high enantioselectivity in Hamashima bromocyclization. In comparison, the Rassias chlocyclization process gave the oxazolines in high optical purity at 5 °C (Table 2), making the method more practical and more appropriate for large scale. However, Hamashima’s catalytic system produces oxazolines of better optical purity.

The enantioselective Toste, Hirokawa, Rassias, and Hamashima halocyclization methods are suitable for amides having an aryl substituent in the styrene position.

**Scheme 7:**
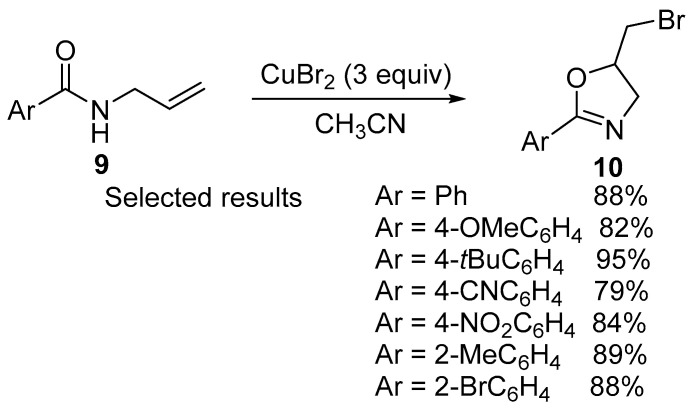
Bromocyclization with utility of CuBr_2_ as bromide source and electrophilic promoter.

A very simple and practical method of bromocyclization involves the use of CuBr_2_ as both the bromide source and the reaction promoter (Scheme 7) [50]. The reaction proceeds under mild, aerobic conditions, producing oxazolines **10** with good yield. A high yield of product was also achieved in the gram-scale experiment.

**Scheme 8:**
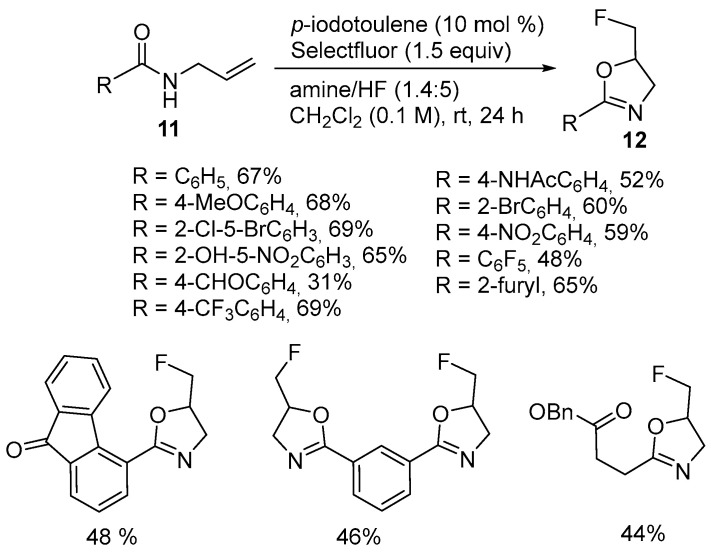
*p*-Iodotoluene-mediated fluorination of *N*-allyl amides.

Organohypervalent iodine compounds have been recognized as efficient oxidative and ligand-transfer reagents in many reactions, including the cyclization of *N*-allyl amides to oxazolines [51]. They have similar reactivity patterns as transition metal catalysts, show excellent oxidizing ability, good stability, and low toxicity. Transformations in the presence of hypervalent iodine compounds are conducted under mild conditions that are tolerated by many functional groups. Among the plethora of hypervalent iodine compounds, (diacetoxyaiodo)arenes and iodosoarenes constitute a group of the most widely used reagents. Often, the catalyst is generated in situ from a precatalyst such as iodobenzene or iodotoluene derivatives. An iodine hypervalent compound-mediated approach to monofluorinated oxazolines **12** was reported by Scheidt (Scheme 8) [52].

**Scheme 9:**
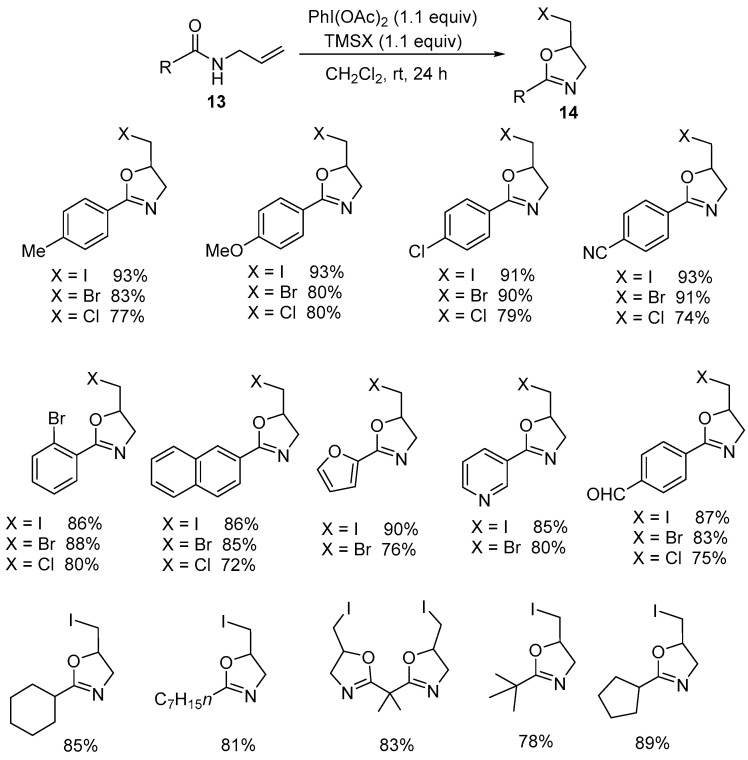
(Diacetoxyiodo)benzene-mediated synthesis of 5-iodo-, 5-chloro-, and 5-bromomethyloxazolines.

According to this procedure, *N*-allyl benzamides **11** treated with a catalytic amount of *p*-iodotoluene as the precatalyst, selectfluor as the oxidant, and amine/hydrofluoric acid in dichloromethane undergoes fluorocyclization to 5-fluoromethyloxazolines **12** in moderate to good isolated yields. Chiral (*S*)-*N*-(1-phenylbut-3-en-2-yl)benzamide under these conditions gave 4-benzyl-5-fluoromethyloxazoline in a yield of 65% and a diastereomeric ratio > 95:5. However, when (*S*)-*N*-allyl-2-(benzyloxy)-3-phenylpropanamide was used as the substrate, no diastereoselectivity was observed, although the reaction proceeded with an excellent yield of over 95%. Using (diacetoxyiodo)benzene as the reaction promoter and halotrimethylsilane as the halogen source, intramolecular cyclization of *N*-allyl amides **13** was performed, leading to the corresponding 5-iodo-, 5-chloro-, and 5-bromomethyloxazolines **14** in good to excellent isolated yields. Structures of some of the obtained halomethyloxazolines **14** are presented in Scheme 9 [53]. A wide range of aromatic and aliphatic amides **13** are reactive under these conditions. The conditions are also applicable for gram-scale synthesis. On the basis of the NMR study, the authors proposed the reaction mechanism.

**Scheme 10:**
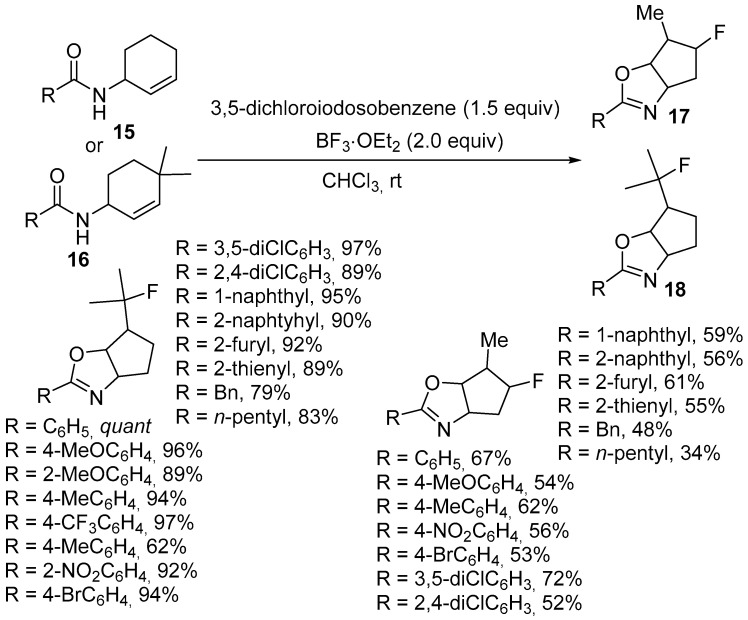
Iodine hypervalent compound-mediated synthesis of five-membered ring-fused monofluorinated oxazolines from *N*-cyclohex-2-enyl allyl amides.

Five-membered ring-fused monofluorinated oxazolines **17** and **18** can be prepared from *N*-cyclohex-2-enyl allyl amides **15** and **16** with BF_3_·OEt_2_ as the fluorine source and 3,5-dichloroiodosobenzene as the iodohypervalent mediator (Scheme 10) [54]. The cyclohexene ring in the substrate undergoes contraction to a monofluorinated five-membered ring. The authors proposed that the mechanism for the ring-contraction monofluorination involves formation of carbocationic intermediates from the *N*-cyclohex-2-enylamides.

**Scheme 11:**
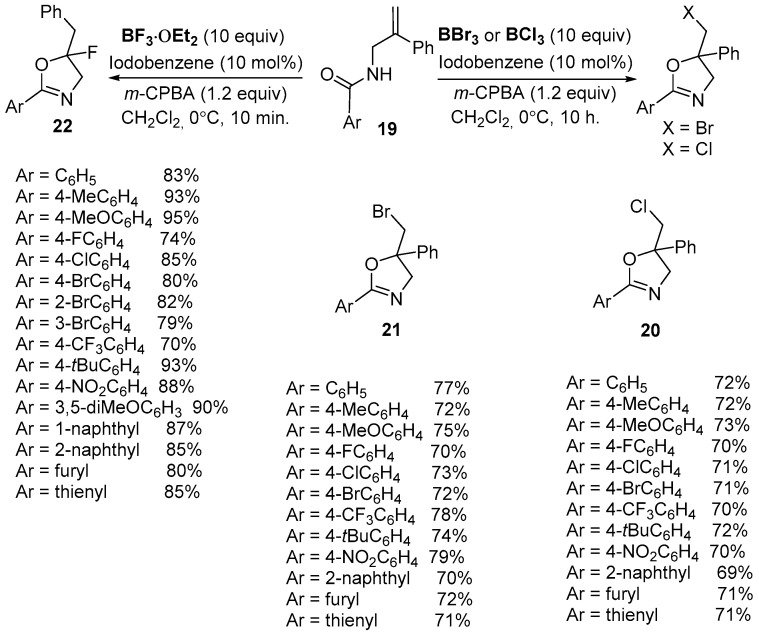
Hypervalent iodine-catalyzed synthesis of 5-halomethyl-2-oxazolines.

Qin and coworkers proposed intramolecular hypervalent iodine-catalyzed halogenation of *N*-allyl amides **19** substituted with phenyl ring in the styrenal position. 5-Chloromethyl- **20** and 5-bromomethyl-2-oxazolines **21** were produced using BCl_3_ and BBr_3_ as chlorinated and brominated reagents, respectively, and iodobenzene as a catalyst precursor in the presence of *meta*-chloroperoxybenzoic acid (Scheme 11) [55]. Various halogenomethylated oxazoline derivatives were synthesized through the catalytic process in high yields. Direct fluorination of the oxazoline ring in position C5 was observed when BF_3_·OEt_2_ as the fluorine reagent was applied under the same conditions (Scheme 11) [56]. Thus, 5-fluoro-2-oxazolines **22** were obtained with a yield of 62–95%. It should be noted that *N*-amides without the phenyl ring at the double bond treated with BF_3_·OEt_2_ underwent hypervalent iodine-catalyzed fluorocyclization to 5-fluoromethyloxazolines **17** and **18** (Scheme 10). All BF_3_, BCl_3_, and BBr_3_ reagents act as a halogen source and activating reagents in the hypervalent iodine(III)-catalyzed halogenations. However, the fluorine source in the fluorination is the [BF_4_]^−^ ion, while the bromine/chlorine source in the bromination/chlorination is the Br^−^/Cl^−^ ion. A detailed mechanistic study undertaken by the authors based on density functional theory calculations explains the difference in the behaviour of BF_3_·OEt_2_, BCl_3_, and BBr_3_ in the intramolecular hypervalent iodine-catalyzed halogenation of *N*-allyl amides [55].

**Scheme 12:**
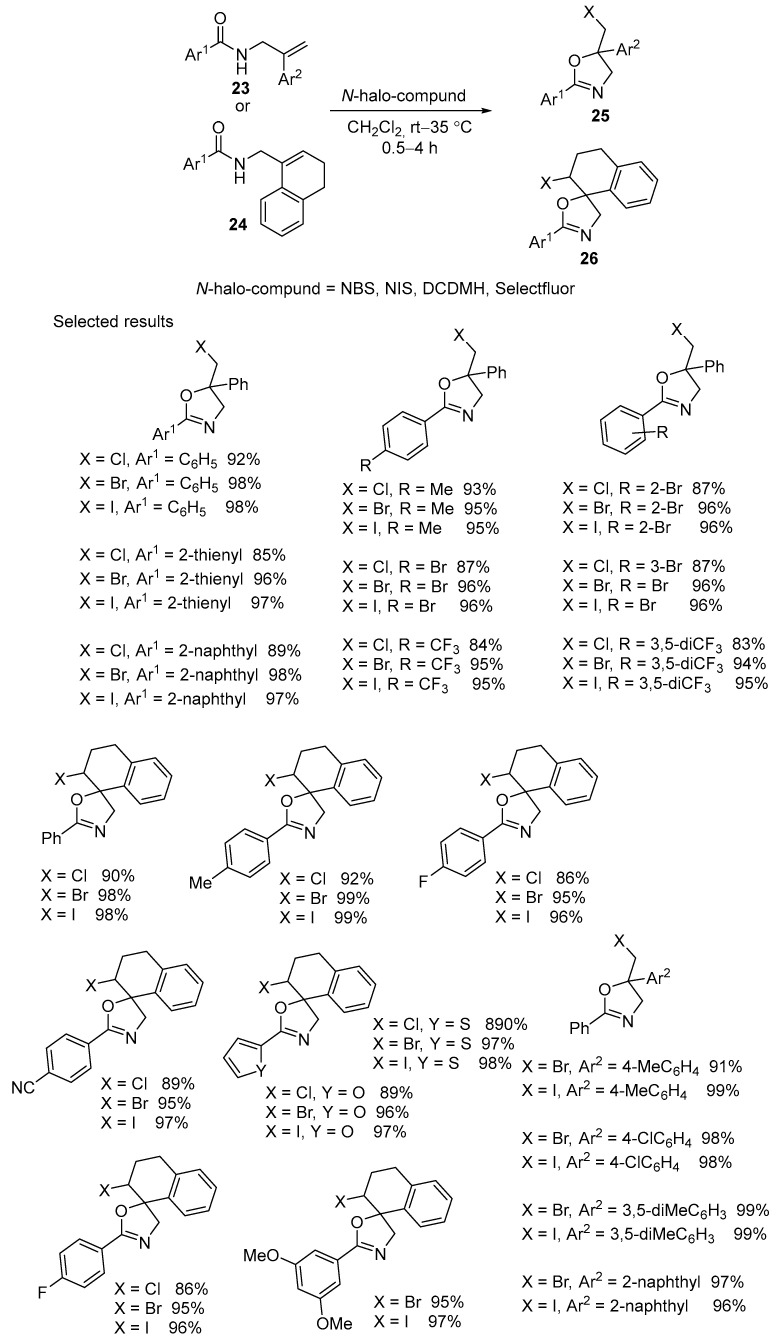
Metal-free synthesis of 5-chloromethyl-, 5-iodomethyl-, and 5-bromomethyl-2-oxazolines.

Metal-free synthesis of 5-chloromethyl-, 5-iodomethyl-, 5-fluoromethyl-, and 5-bromomethyl-2-oxazolines **25** including spiro derivatives **26** was reported using DCDMH, NIS, Selectfluor, and NBS, respectively, as the halogen source and oxidants (Scheme 12) [57]. The advantage of this method is that mild reaction conditions are required, and a metallic catalyst does not have to be used to obtain the halogenated oxazolines in high to excellent isolated yields (up to 99%).

**Scheme 13:**
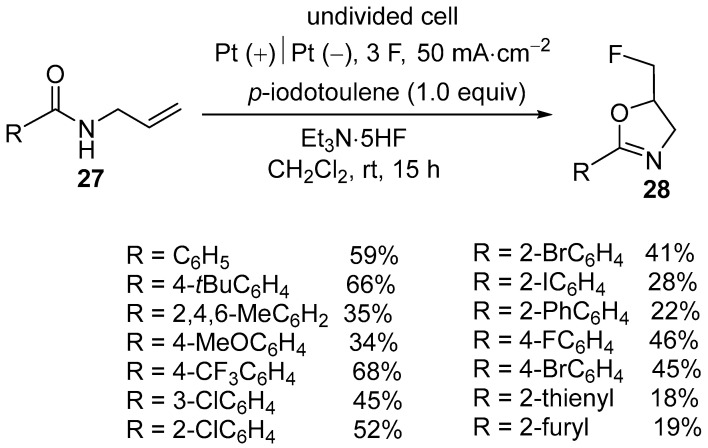
Electrochemical *p*-iodotoluene-mediated fluorination of *N*-allyl amides.

Hypervalent iodine catalysis has many advantages. However, it is accompanied by a low atom economy and the production of large amounts of organic waste. To overcome these issues, Haupt developed an electrochemical version of hypervalent iodine-catalyzed fluorocyclization, allowing for the synthesis of 5-fluoromethyl-2-oxazolines **28** with moderate yields (Scheme 13) [58]. In contrast to conventional methods, the electrochemical oxidation of iodoarene to the hypervalent iodine species eliminates the need to use an oxidizer. Therefore, electrochemical methods are consistent with the principles of green chemistry.

**Scheme 14:**
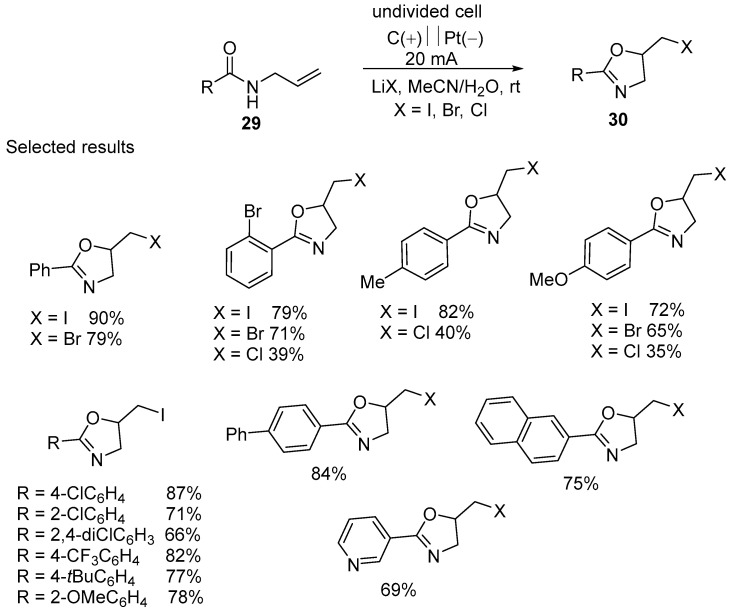
Electrochemical oxidative cyclization of *N*-allylcarboxamides to iodo-, bromo-, and chloromethyloxazolines.

In 2022, the utility of lithium halides as a redox medium and electrolyte in another electrochemical oxidative cyclization of *N*-allyl amides **29** to iodo-, bromo-, and chloromethyl oxazolines **30** was described (Scheme 14) [59]. The process was carried out in an undivided cell with a platinum electrode as the cathode and a graphite rod as the anode. A broad scope of 5-halomethyloxazolines **30** were formed under this condition with good yields and selectivity.

### 2.2. Trifluoromethyl Functionalized Oxazolines

**Scheme 15:**
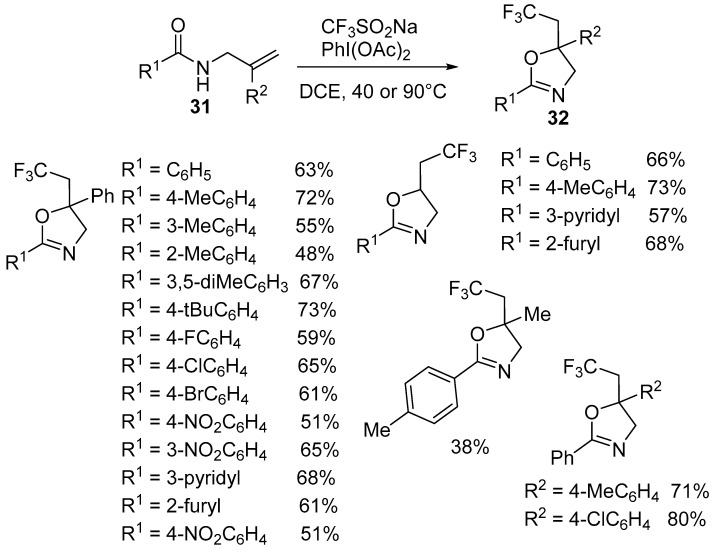
Trifluoromethylation of 4-methyl-*N*-(2-phenylallyl)benzamide using Langlois reagent (CF_3_SO_2_Na).

Trifluoromethylation of *N*-benzamide **31** using the Langlois reagent (CF_3_SO_2_Na) and (diacetoxyiodo)benzene as an oxidant led to the formation of trifluoromethylated oxazolines **32** in moderate to good yields (Scheme 15) [60].

**Scheme 16:**
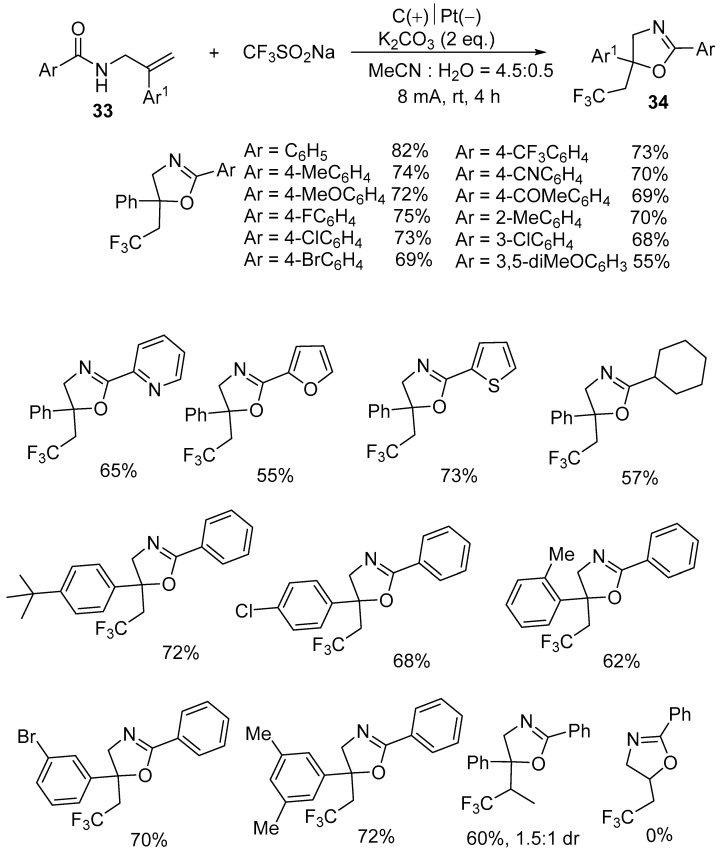
Electrochemical synthesis of trifluoromethylated oxazolines.

A green protocol for the electrochemical method for the synthesis of trifluoromethylated oxazolines using the Langlois reagent (CF_3_SO_2_Na) as a trifluoromethyl source has been reported [61]. According to this protocol, a series of *N*-allyl amides **33** were subjected to a reaction with CF_3_SO_2_Na, using K_2_CO_3_ as the base and a mixture of MeCN/H_2_O (4.5:0.5) as the cosolvent (Scheme 16). The reaction was conducted under constant current (8 mA) electrolysis conditions, using a graphite rod anode and a platinum plate cathode that led to trifluoromethylated oxazolines **34** in moderate to good yields. The scope of amides includes benzamide derivatives that are substituted with either electron-accepting or electron-donating groups, as well as heterocyclic amides. The cyclohexane-2-amide derivative was also found to be reactive in this reaction, producing the desired oxazoline in a yield of 57%. The presence of a phenyl ring in the vinyl position of the amide, whether substituted or not, appeared to be crucial for the outcome of the reaction. The reaction with simple *N*-allylbenzamide did not proceed.

**Scheme 17:**
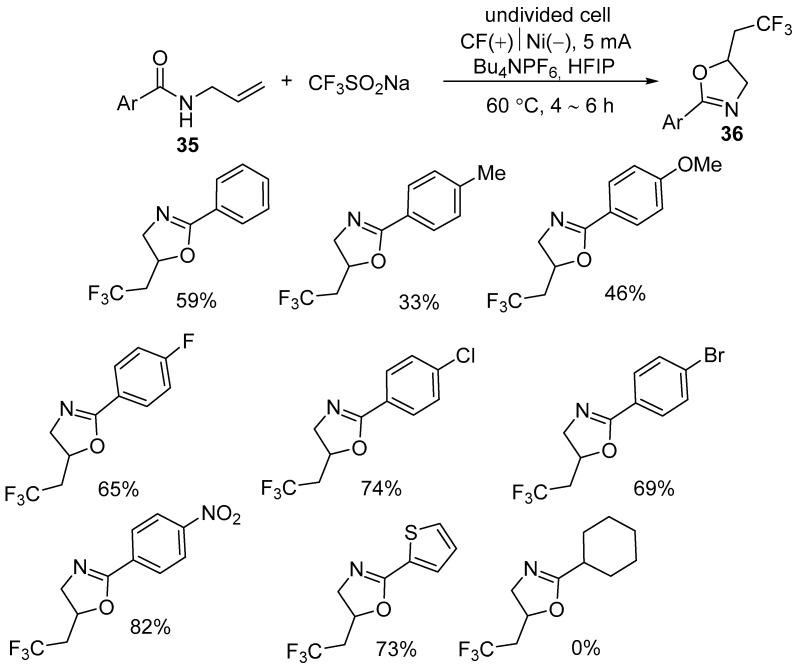
Electrochemical synthesis of trifluoromethylated oxazolines using CF_3_SO_2_Na in the presence of Bu_4_NPF_6_.

An alternative electrochemical method for the construction of trifluorinated oxazolines was described. This method uses cheap carbon fibre as the anode and nickel plates as the cathode in an undivided cell (Scheme 17) [62]. No external chemical oxidants, metal catalysts, or additives are required. *N*-allylbenzamides **35** with various substituents on the phenyl ring such as the methyl, methoxy, halides, and nitro groups reacted smoothly with CF_3_SO_2_Na in HFIP in the presence of Bu_4_NPF_6_ as an electrolyte under a constant current of 5 mA, producing oxazolines **36** with a 33–82% yield. *N*-allylfuran-2-carboxamide was also a suitable substrate for this process in contrast to the aliphatic aldehyde, cyclohexane-2-carboxyamide, which proved to be non-reactive at these electrochemical conditions. A possible mechanism for the reaction is also proposed.

**Scheme 18:**
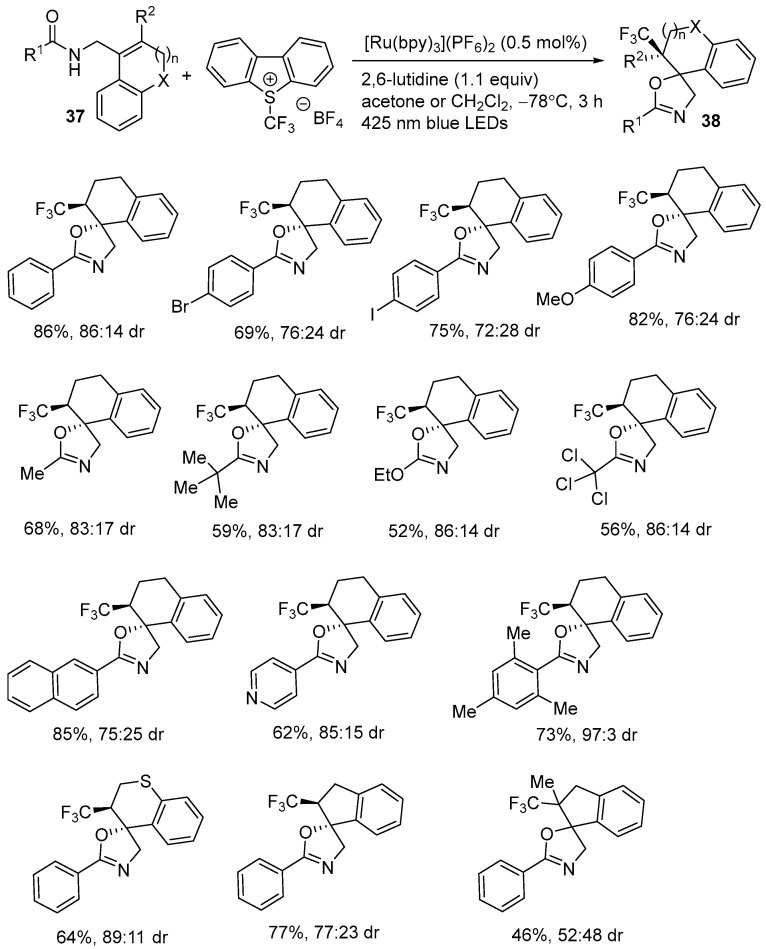
Photocatalytic synthesis of trifluoromethylated spirooxazolines.

**Scheme 19:**
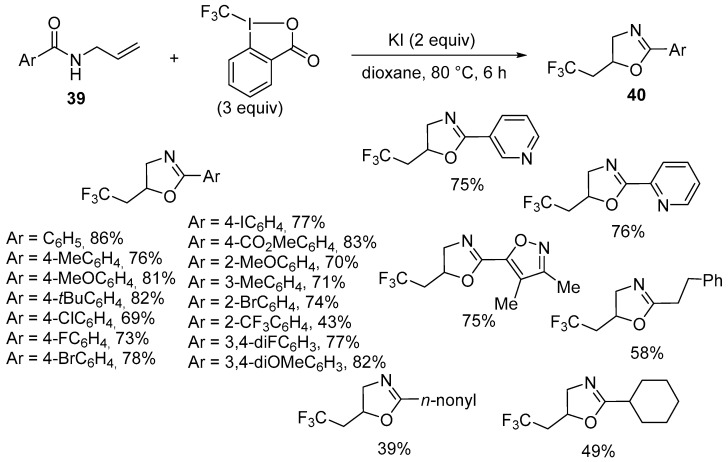
Synthesis of trifluoromethylated oxazolines using the Togni reagent.

**Scheme 20:**
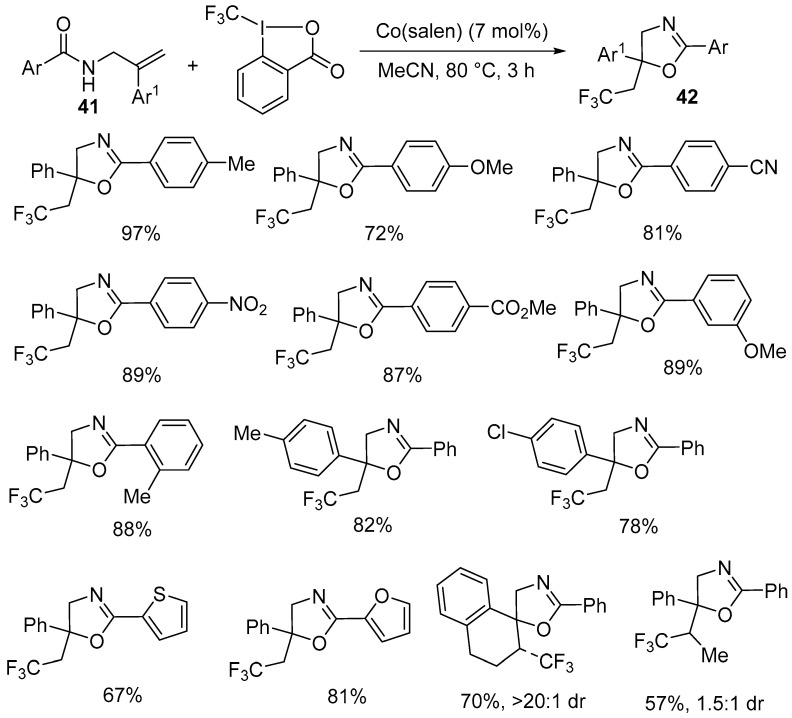
Cobalt-catalyzed oxytrifluoromethylation of *N*-allyl amides.

The well-known photocatalyst [Ru(bpy)_3_]^2+^ and Umemoto’s electrophilic trifluoromethylating reagent were adopted for regiospecific trifluoromethylative spirocyclization (CF_3_-spirocyclization) of cyclic *N*-allyl amides **37** (Scheme 18) [63]. The photocatalytic system enables the diastereoselective synthesis of CF_3_-containing spirooxazolines **38** from various amides with different cyclic alkene groups. The amide with a bulky 2,4,6-trimethylphenyl ring gave the corresponding oxazoline in a 73% yield with excellent diastereoselectivity (97:3 dr). No diastereoselctivity was observed in the reaction of [(1*H*-2-methylinden-3-yl)methyl]benzamide. Diastereoselectivity is thus dependent on the substituent of the amide group and the structure of the cyclic alkenes. Kawamura proposed a simple and efficient method for the synthesis of trifluoromethylated oxazolines using the Togni reagent in the presence of KI (Scheme 19) [64]. Reactions were carried out in dioxane at 80 °C and produced oxazolines **40** in good yields. Various substituents on the aromatic ring of *N*-allyl arylamide **39** were well tolerated under mild reaction conditions. The utility of the methodology was proved by applying two amides as substrates obtained from bioactive telmisartan and lithocholic acid. In both reactions, respective 5-trifluoromethyloxazolines were formed in 51 and 35% yields. Recently, a cobalt-catalyzed oxytrifluoromethylation of *N*-allyl amides **41** for the straightforward synthesis of various CF_3_-containing oxazolines **42** with Togni reagent as the trifluoromethyl precursor under mild reaction conditions has been developed (Scheme 20) [65]. In the presence of 7 mol% of Co(salen) in acetonitrile at 80 °C, the desired oxazolines **42** containing the CF_3_ moiety were obtained in a yield of 57–97%. The substrate scope is limited to benzamide and heteroaryl amide derivatives since alkyl amides are not tolerated in these conditions. The authors propose that the CF_3_-containing oxazolines were formed via a radical–cation crossover mechanism.

The methods of trifluoromethylation of *N*-allyl amides are summarized in Table 3. The highest yields for this transformation were reported using the Co(salen)-catalyzed system (Table 3, entry 6, and Scheme 20). However, the main limitation of this methodology is that it is not applicable to aliphatic amides. This is in contrast to Ru-catalyzed cyclization, which tolerates aliphatic amides well (Table 3, entry 4, and Scheme 18). The latter method, on the other hand, is limited by the need to synthesize the appropriate amide substrates. Although the application of electrochemistry to the trifluoromethylation of *N*-allyl amides did not significantly increase the process yield compared to conventional methods (see Table 3, entries 2 and 3), it is appealing from a green chemistry standpoint (Table 3, entry 2 and 3).

### 2.3. Sulfonyl Funtionalized Oxazolines

**Scheme 21:**
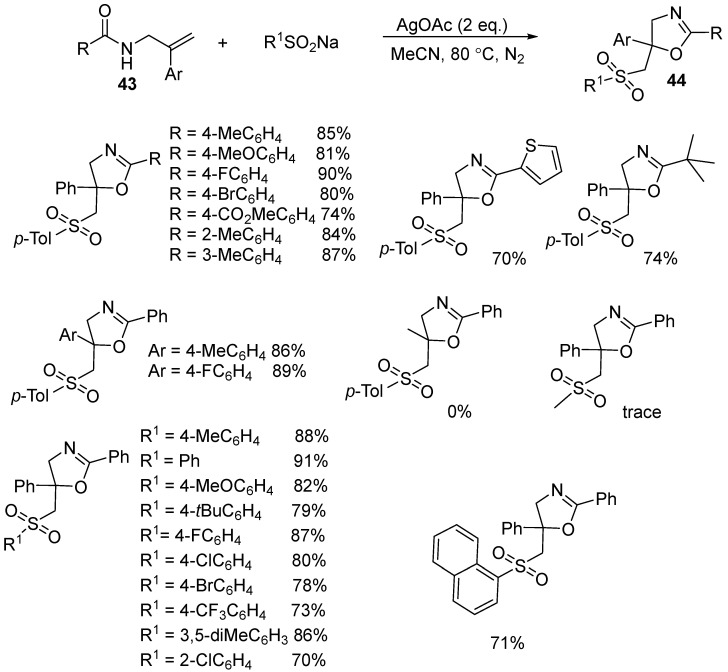
Silver-mediated radical cyclization of *N*-s into sulfonated oxazolines.

Silver-mediated radical cascade cyclization of *N*-allyl amides **43** into sulfonated oxazolines **44** involving sodium sulfinates was elaborated on (Scheme 21) [66]. In the presence of two equivalents of silver acetate, sodium sulfinate undergoes addition to the alkene moiety of the amide, followed by intramolecular cyclization providing sulfonated oxazolines with the yield of 60–91%. The process is applicable to the synthesis of a wide range of sulfonated oxazolines **44** using a variety of amides **43** and arylsulfinates. The scope of reactive sulfinates includes benzenesulfinate and its derivatives with various substituents in the benzene ring, naphthalene-1-sulfinate, and thiophene-derived sulfinate. However, the range of amides is limited to derivatives of benzamide and thiophene-2-carboxamide that are substituted on a carbon–carbon double bond with a benzene ring.

**Scheme 22:**
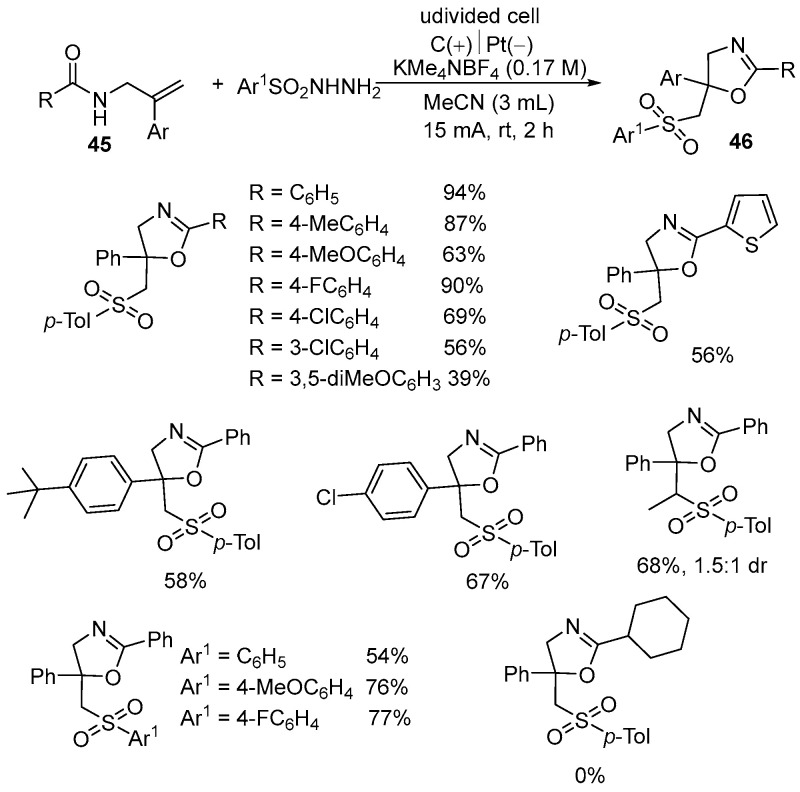
Electrochemical synthesis of sulfonated oxazolines from allyl amides and ArSO_2_NHNH_2_.

**Scheme 23:**
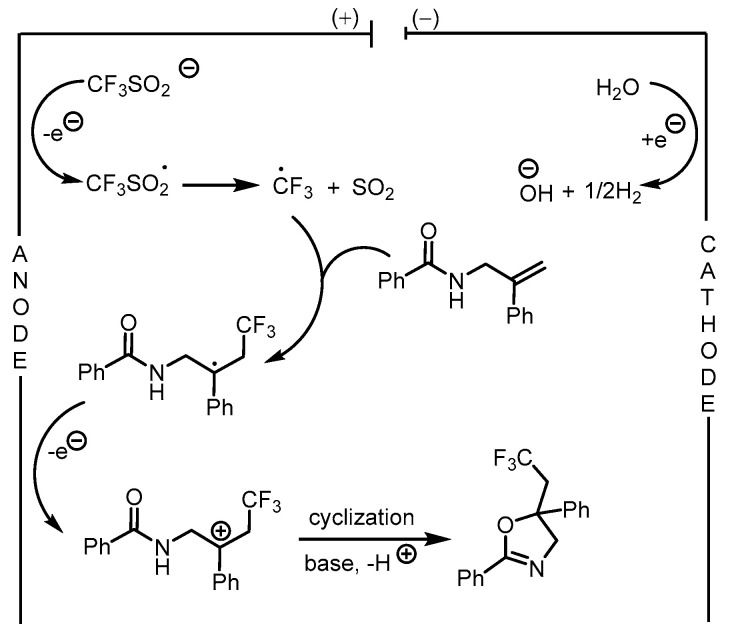
Mechanism of the electrochemical cyclization of **45**.

Huang and co-workers proposed an electrochemical method for the synthesis of sulfonated oxazolines **46** from allyl amides **45** using ArSO_2_NHNH_2_ as a sulfonyl source (Scheme 22) [61]. The electrochemical process took place under constant current electrolysis conditions employing a graphite rod anode and platinum plate cathode with Me_4_NBF_4_ as an electrolyte and MeCN as a solvent. A variety of benzaldehyde derivatives with electron-withdrawing (F, Cl) or electron-donating (OMe, Me) substituents in the benzene ring reacted smoothly to produce the corresponding sulfonated oxazolines in good yields. Furthermore, the benzaldehyde with a 3,5-disubstituted OMe group at the benzene ring, as well as thiophen-2-amide, were effective in this electrochemical system. Benzaldehyde with an internal double bond also successfully produced the corresponding oxazoline with 1.5:1 dr. On the contrary, the only aliphatic amide tested, cyclohexane-2-carboxamide, appeared to be unreactive under these conditions. Benzenesulfonyl hydrazine and its derivatives with the OMe group and fluorine atom in the *para*-position of the benzene ring were found to be an active sulfonyl sources. Two radical trapping experiments were undertaken to prove the radical nature of the process. It was also demonstrated that the sulfonyl radical was involved in this process (Scheme 23). The method shows potential practical utility, since the scale-up experiment produced the appropriate oxazoline in good yield.

**Scheme 24:**
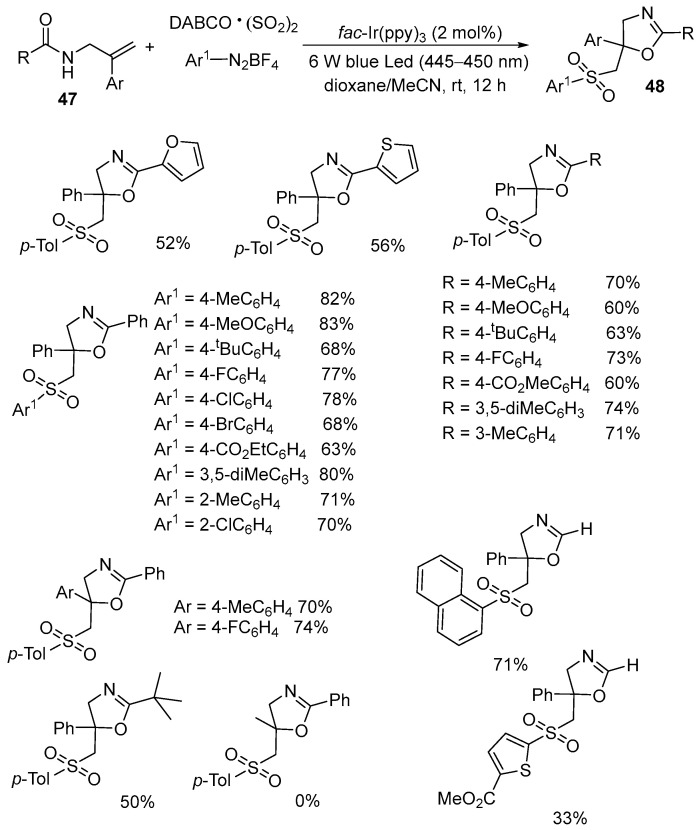
Photoredox-catalyzed cyclization of *N*-allyl amides.

A range of sulfonated oxazolines **48** was obtained in moderate to good yields in the photoredox-catalyzed cyclization of *N*-allyl amides **47** elaborated by Cui and coworkers (Scheme 24) [67]. This is a three-component reaction of *N*-allyl amides, aryldiazonium salts, and DABCO·(SO_2_)_2_, which serves as a sulfonyl source, conducted under mild reaction conditions. The transformation involves the sequential insertion of sulphur dioxide, intermolecular sulfonylation of alkenes, and intramolecular cyclization via a radical mechanism. Both the photocatalyst *fac*-Ir(ppy)_3_ and the visible light emitted by the 6W blue LEDs (445–450 nm) were essential for the reaction to proceed. Diverse aromatic, heteroaromatic, and aliphatic aldehydes were shown to react excellently, giving rise to the oxazolines in the good yield. Only amides with a phenyl substituent in the vinyl position were active in the reaction. The replacement of the phenyl ring with a methyl group made the amide unreactive. Aryldiazonium tetrafluoroborates differently substituted on the aromatic ring were suitable substrates to provide sulfonated oxazolines in 60–83% yields.

Naphthalene-1-diazonium and tiophene-2-diazonium tetrafluoroborates also appeared to be reactive in the process; however, the yield observed in the reaction with the latter salt was low. It is suggested that a radical mechanism is involved in this reaction, and arylsulfonyl free radicals were generated in situ from DABCO·(SO_2_)_2_ and aryldiazonium salts. The synthetic utility of the methodology was proved by successfully conducting a scale-up experiment.

Sulfonated heterocycles are recognized as biologically important compounds that exhibit unique properties. Thus, the synthetic methods summarized in this chapter may be of interest to both academic and industrial communities. The silver-catalyzed method offered the most promising results in terms of yield (Table 4, entry 1). In this system, both aromatic and aliphatic amides are active. Aliphatic amides are also tolerated in photoredox-catalyzed cyclization (Table 4, entry 3). However, the cyclization of aliphatic amides produces oxazolines in a lower yield.

### 2.4. Sulfenyl-Functionalized Oxazolines

**Scheme 25:**
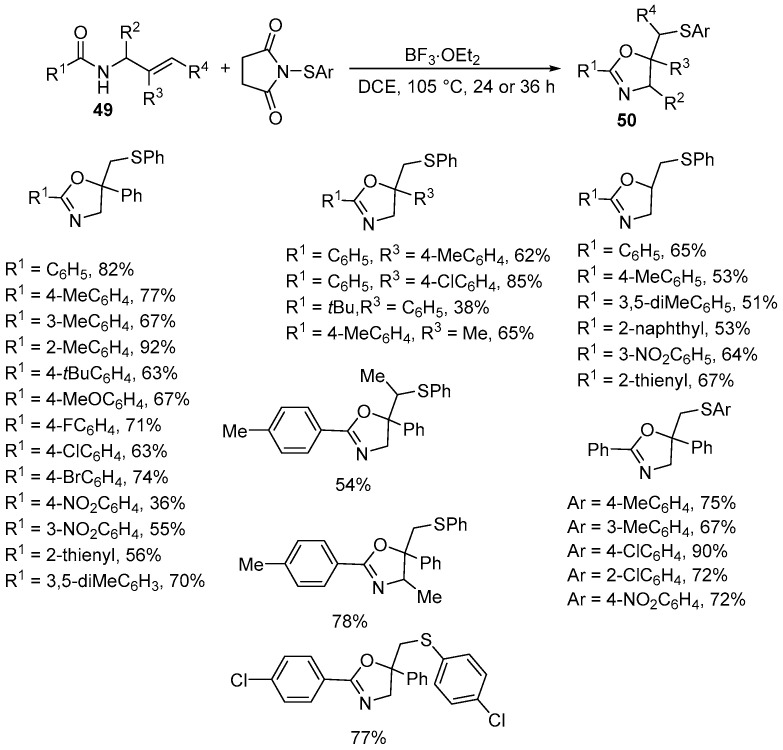
Boron-activated arylsulfenylation of *N*-allyl amides with PhS-succinimide.

Research on obtaining arylsulfenyl derivatives of oxazoline by the cyclization of unsaturated amides was also elaborated upon. Boron-activated arylsulfenylation of *N*-allyl amides **49** with PhS-succinimide gave the arylsulfenyl-substituted oxazolines **50** in moderate to good yields (Scheme 25) [68]. Crucial for the reaction rate was the nature of the R^3^ substituent. Amides with aromatic rings as R^3^ underwent cyclization faster than those substituted with methyl groups or without an R^3^ substituent. This method seemed simple and practical. The approach uses inexpensive 1-(arylsulfanyl)pyrrolidine-2,5-diones as the sulfenyl source and boron trifluoride etherate as the catalyst.

**Scheme 26:**
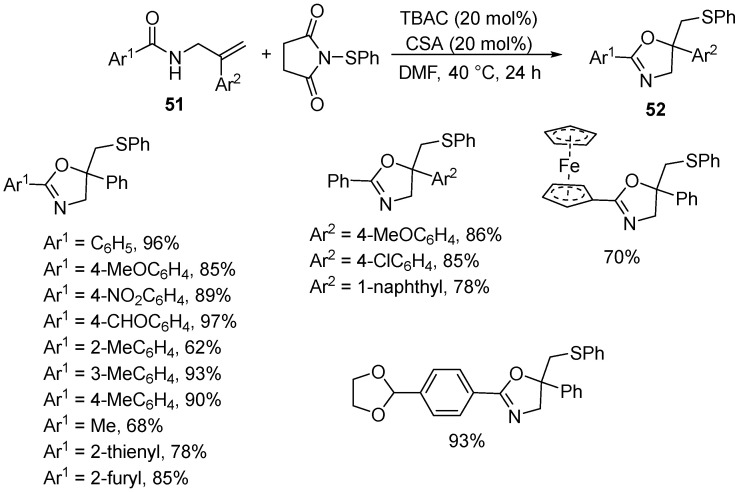
Brønsted acid/tetrabutylammonium salt-catalyzed synthesis of 5-[(arylsulfenyl)methyl]oxazolines.

Another method is a Nagao-elaborated catalytic system composed of 10-camphorsulfonic acid (CSA) and tetrabutylammonium chloride (TBAC) as an activator to perform cyclization of *N*-allyl amides **51** into 5-[(arylsulfenyl)methyl]oxazolines **52** with PhS-succinimide as an electrophile and source of sulfenyl moiety (Scheme 26) [69]. This method enabled the synthesis of 5-[(arylsulfenyl)methyl]oxazoline **52** derivatives in good yields. In these conditions, amides with Ar^2^ = Me or H were active and gave corresponding arylsulfenyloxazolines in a yield of 73% and 72%, respectively. Scaling up the reaction to the gram scale afforded the product without a significant loss in yield. The advantage of this reaction is that it proceeds at a low temperature in comparison to the boron trifluoride etherate-catalyzed reaction discussed above.

**Scheme 27:**
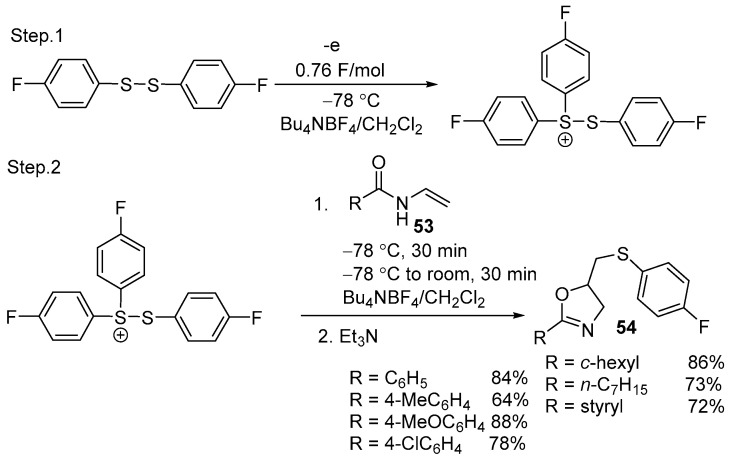
Two step synthesis of arylsulfenyl oxazolines from ArSSAr.

The arylsulfenyl derivatives of oxazoline **54** can be prepared from *N*-allyl amides **53** and Ar(ArSSAr)^+^ in two-step synthesis (Scheme 27) [70]. In the first step, ArS(ArSSAr)^+^BF_4_^−^ is electrochemically generated from ArSSAr in Bu_4_NBF_4_/CH_2_Cl_2_. The second step consists of typical cyclization initiated by the electrophilic addition of ArS(ArSSAr)^+^ to the double carbon–carbon bond. This approach allowed the preparation of various 5-arylsulphenyloxazolines **54** in good yields (Scheme 27).

**Scheme 28:**
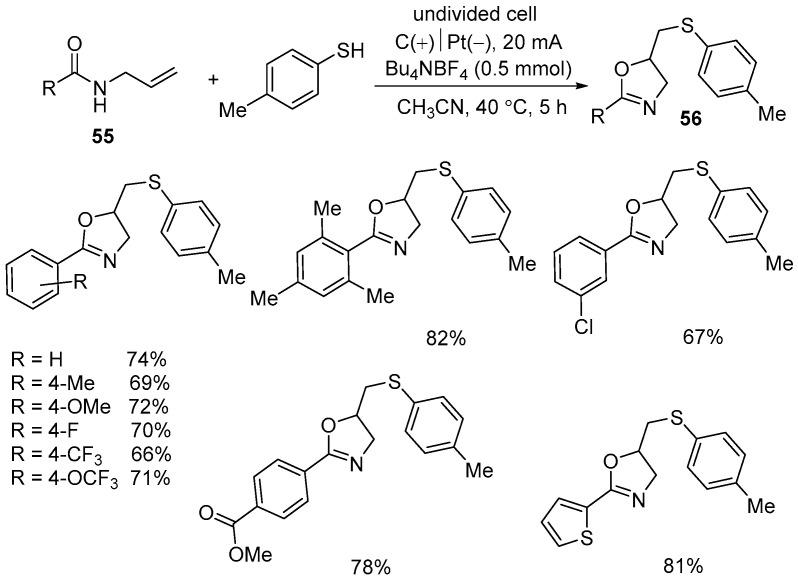
Electrochemical intramolecular cyclization with 4-methylbenzenethiol.

Similar electrochemical intramolecular cyclization with 4-methylbenzenethiol as an electrophile also gave the arylsulfenyl oxazolines **56** in good yields (Scheme 28) [71].

These two-latter, green chemistry-friendly electrochemical reactions proceed smoothly without the addition of an external oxidizing agent or metallic catalyst. In addition, the two-step method was proven to be applicable for the cyclization of aliphatic *N*-allyl amides.

**Scheme 29:**
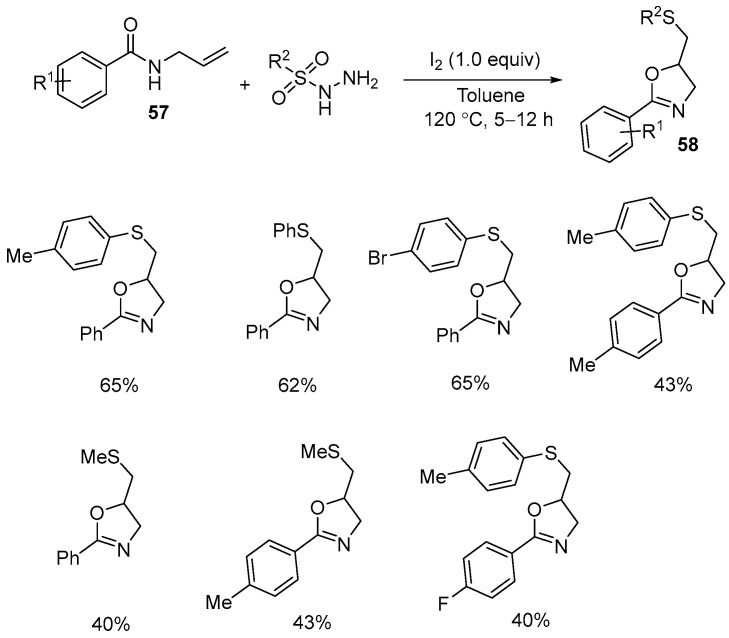
Sulfenylation of *N*-allyl amides with sulphonylhydrazides in the presence of iodine.

Surprisingly, the sulfenylation took place when *N*-allyl amides **57** were treated with sulphonylhydrazides in the presence of iodine (Scheme 29) [72]. The formation of sulfonyl derivatives was not observed in the reaction. The authors described a plausible mechanism for the formation of oxazolines. They suggested the formation of ArS-I species as the sulfenyl group source and the active electrophile as responsible for the activation of the carbon–carbon double bond.

**Scheme 30:**
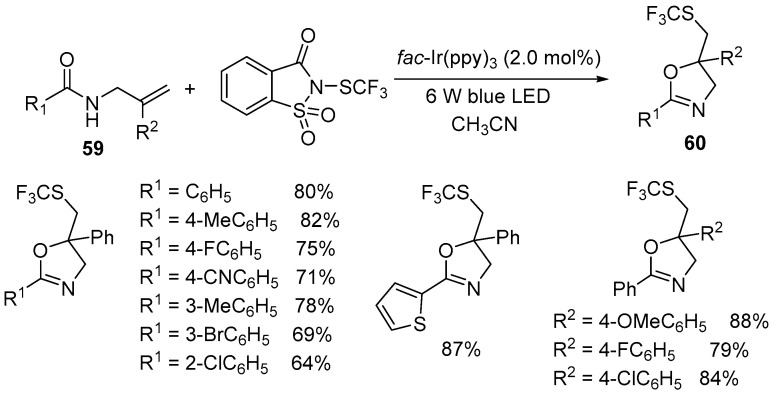
Visible light-induced photocatalytic trifluoromethylthiolation/cyclization of *N*-allyl amides.

The trifluoromethylthio (SCF_3_) group is present in numerous biologically active compounds. Taking that fact into account, the chemoselective incorporation of the trifluoromethylthio (SCF_3_) group into the oxazoline molecule has been performed using *N*-trifluoromethylthiosaccharin as a SCF_3_ source and *fac*-Ir(ppy)_3_ as the photocatalyst under 6 W blue LED irradiation (Scheme 30) [73]. The reactions with a variety of *N*-allyl amides **59** proceeded smoothly to produce the oxazolines **60** in good yields.

Table 5 summarizes the synthetic methods of sulfenyl functionalized oxazolines. A diverse range of thio derivatives can be used as a source of the sulfenyl moiety. Sulfonohydrazines also yielded sulfenyl derivatives, but with significantly lower yields (Table 5, entry 5).

### 2.5. Selenyl-Functionalized Oxazolines

**Scheme 31:**
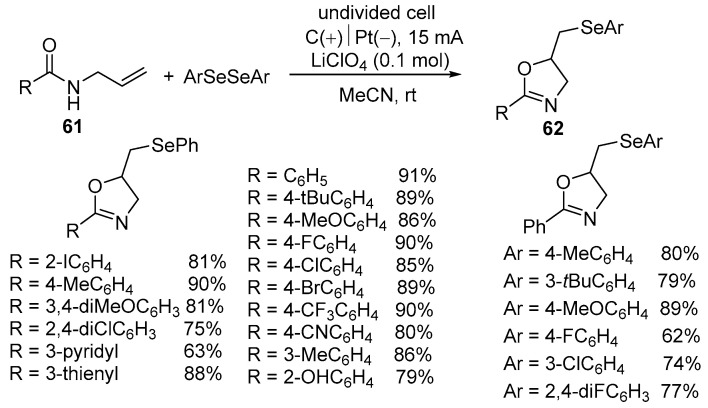
Electrolytic selenocyclization of *N*-allyl amides.

The C-Se bond is an essential structural motive found in many bioactive molecules and natural products. Furthermore, the selenium group can be modified to other moieties, which makes it important from a synthetic point of view. Thus, the selenylation/cyclization of *N*-allyl amides can give biologically interested selenofunctionalized oxazolines. Sarkar subjected *N*-allyl benzamides **61** and diaryl diselenides to electrolysis at a constant current of 15 mA. The electrolysis was conducted in acetonitrile containing 0.1 M LiClO_4_ in an undivided cell equipped with a platinum cathode and a graphite anode (Scheme 31) [74]. Under these conditions, a series of selenated oxazolines **62** was obtained with an isolated yield of 62–91%.

**Scheme 32:**
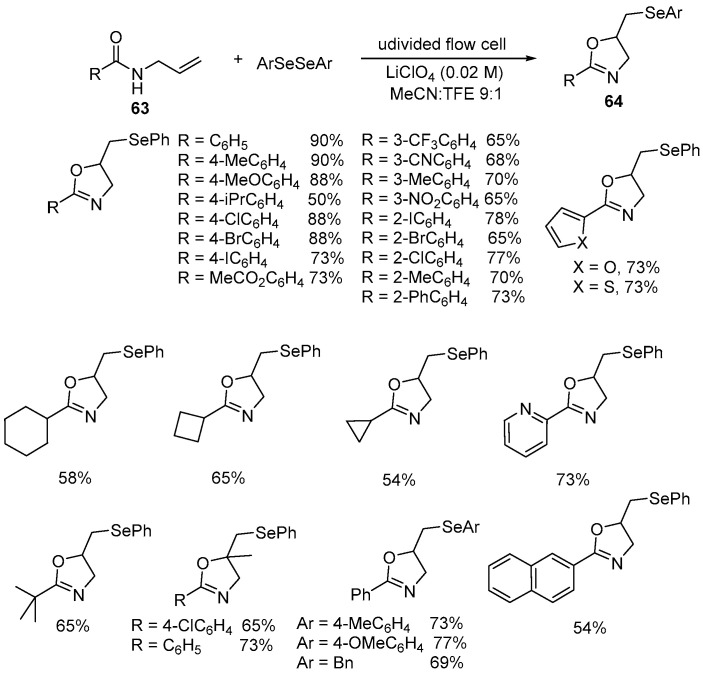
Flow electrochemical selenocyclization of *N*-allyl benzamides.

Another electrochemical approach to the selenocyclization of *N*-allyl benzamides to selenofunctionalized oxazolines uses continuous flow electrochemistry (Scheme 32) [75]. Using a graphite electrode as the anode and as the cathode solution of *N*-allylbenzamide **63** and diaryl diselenide in the presence of a small amount of LiClO_4_ in a mixture of acetonitrile and 2,2,2-trifluoroethanol was pumped with a flow rate of 0.15 mL min^−1^ and was electrolyzed under constant current conditions. Seventeen *N*-allyl benzamide derivatives **63** undergo selenocyclization to oxazolines **64** with good to excellent yields, regardless of the nature and position of the substituent in the phenyl ring. The method appeared to be applicable for heterocyclic and aliphatic *N*-allyl amides as well. On the other hand, the application of 1.61 g of *N*-allyl benzylamide to the flow electrochemical conditions ended up with a lower yield due to some fouling of the electrode, which is visible after 8 h of reaction time.

**Scheme 33:**
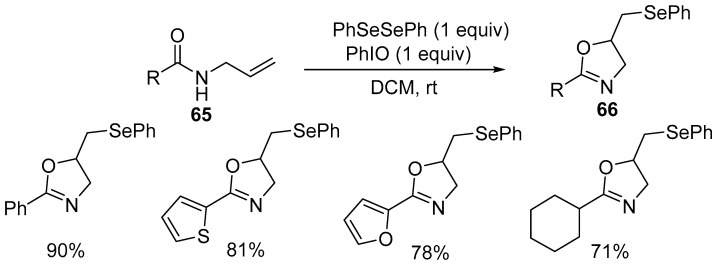
Selenocyclization of *N*-allyl amides mediated by hypervalent iodine (III) catalyst (PhIO).

An intramolecular selenocyclization of *N*-allyl amides **65** mediated by a commercially available hypervalent iodine (III) catalyst (PhIO) has been developed (Scheme 33) [76]. The method involves treating the amide with diphenyl diselenide in the presence of one equivalent of PhIO in dichloromethane at room temperature. This method provides access to a few selenenylated oxazolines **66** in yields of 71–90%.

In summary, the electroselenocyclization methods have a fairly wide range of applications. The scope of the Sakar electrochemical method is quite broad (Table 6, entry 1, and Scheme 31) but limited to *N*-allyl benzamide and *N*-allyl heteroaromatic amide derivatives. Reactions of aliphatic amides were not conducted under Sakar conditions. The flow electrochemical methodology is very attractive due to the wide range of amide substrates that are active in the reaction (Table 6, entry 2, and Scheme 32). However, the difficulty of adapting the method to the gram scale is a serious limitation of this approach. Further efforts are needed to expand the scope of the hypervalent iodine-catalyzed reaction to include a wider range of amides. Only four oxazolines were synthesized under these catalytic conditions (Table 6, entry 3, Scheme 33). So far, the only selenating agents described in the literature are derivatives of diaryl diselenide.

### 2.6. Acetoxy-, Hydroxyl-, Amino-, and Alkyl-Functionalized Oxazolines

**Scheme 34:**
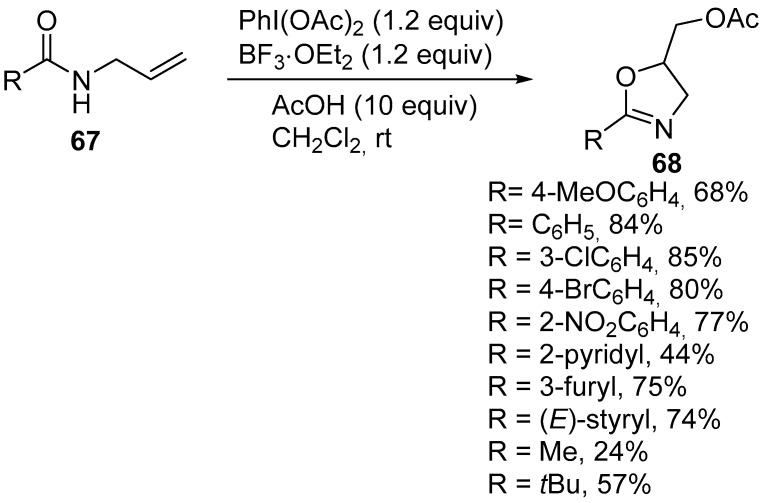
Synthesis of acetoxymethyloxazolines mediated by hypervalent iodine (III) catalyst.

Horn applied (diacetoxyiodo)benzene in the presence of BF_3_·OEt_2_ and acetic acid to obtain several 5-acetoxy oxazolines **68** in good yields (Scheme 34) [77].

**Scheme 35:**
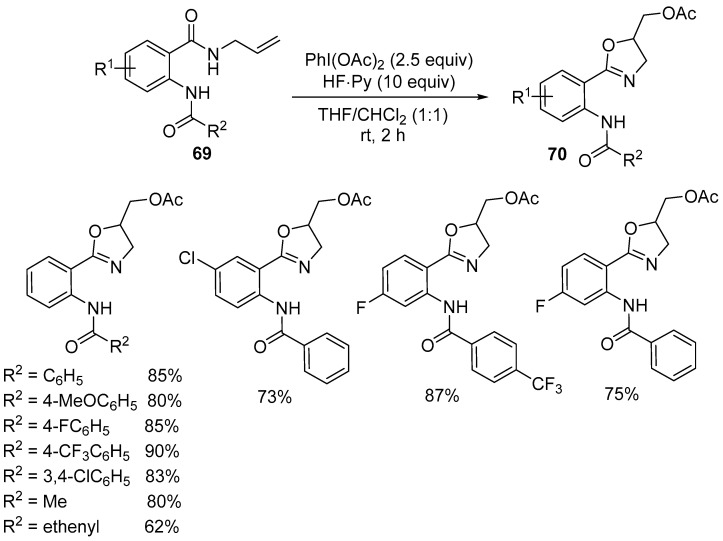
Cyclization of *N*-allyl amides mediated by (diacetoxyiodo)benzene/hydrogen-fluoride-pyridine.

Intramolecular cyclization of *N*-allyl amides **69** mediated by the system (diacetoxyiodo)benzene/hydrogen-fluoride-pyridine furnished the respective acetoxymethyloxazolines **70** in moderate to good yields (Scheme 35) [78]. The use of substituted *N*-(*E*)-allylamides with an aryl ring at the distal end of the olefin causes a change in regioselectivity and the formation of the oxazine *endo*-product was observed. This is due to the formation of a favourable carbocation stabilized by the additional aryl ring.

**Scheme 36:**
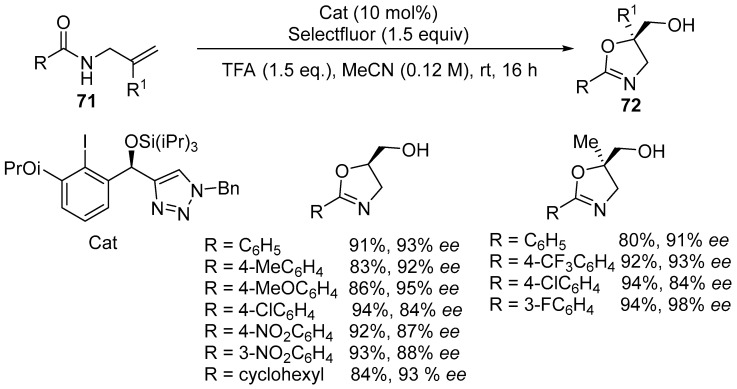
Enantioselective synthesis of hydroxymethyloxazolines mediated by hypervalent iodine (III) catalyst.

**Scheme 37:**
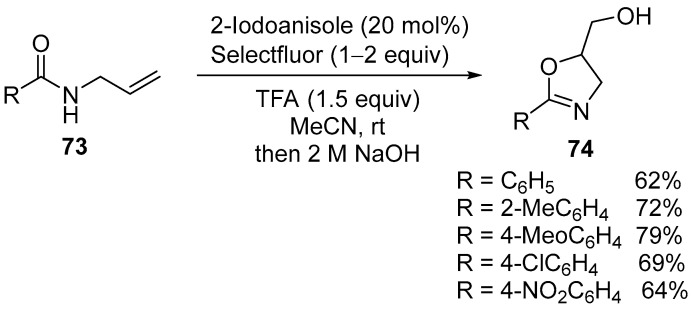
Synthesis of hydroxymethyloxazolines.

Nachtsheim and his group developed a C1-symmetric chiral iodoarene substituted with triazole, in which triazole acts as a direct stabilizing donor of the hypervalent iodine centre through a dative N−I interaction (Scheme 36) [79]. The catalyst was applied to the enantioselective intramolecular oxycyclization of several *N*-allyl amides **71**. Reactions carried out in the presence of TFA and Selectfluor as co-oxidant in acetonitrile after quenching with water provided the corresponding hydroxymethyloxazolines **72** in high yields (80–94%) and excellent enatioselectivities, ranging from 84 to 98% (Scheme 36). The mechanism of the process was proposed by the authors. Chiral oxazolines that possess the hydroxyl group in the alkyl chain constitute important intermediates in organic synthesis. It was demonstrated that they can be converted into other chiral oxazoline derivatives, amino alcohols, and amides. Moran elaborated the catalytic oxidative cyclization of *N*-allyl amides **73** using hypervalent iodine species generated in situ. 2-Iodoanisole has been found to be the best precatalyst compared to other iodoarenes tested in this reaction (Scheme 37) [80].

The methodology that combines Selectfluor as an oxidant, and trifluoroacetic acid and acetonitrile as a solvent allowed hydroxymethylated oxazolines **74** to be produced in moderate to good yields (Scheme 37). Amides possessing alkyl groups at the C-C double bonds were also well tolerated. In contrast, trisubstituted and aryl-substituted substrates did not undergo cyclization. The effect of the iodoarene precatalyst on the reaction rate and mechanism of the cyclization based on DFT modelling has been described [81].

**Scheme 38:**
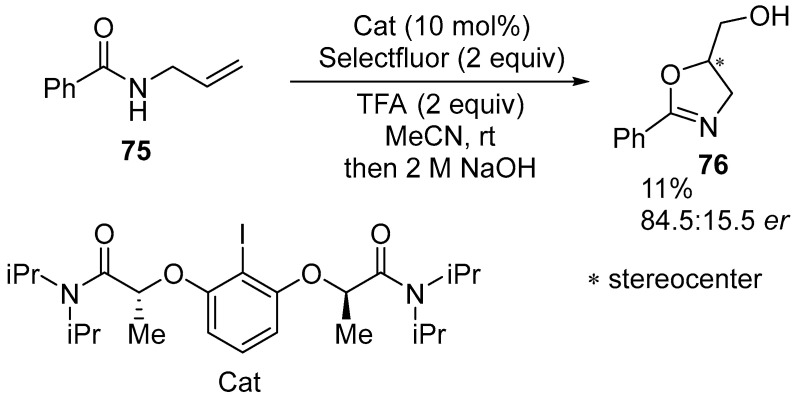
Enantioselective synthesis of hydroxymethyloxazolines.

Phenyllactamide-based C2-symmetric chiral organoiodine catalyst (Scheme 38) was adopted to elaborate an enantioselective version of the catalysis. Its application to the cyclization of *N*-allyl benzamide **75** led to corresponding oxazoline **76** in a yield of only 11% and an enantiomeric ratio of 84.5:15.5.

**Scheme 39:**
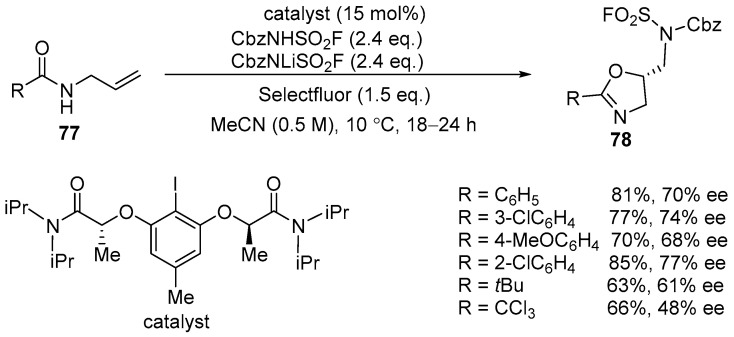
Stereoselective intramolecular oxyamination of *N*-allyl amides.

This catalyst was applied with better success to the stereoselective intramolecular oxyamination of *N*-allyl amides **77** by Hashimoto (Scheme 39) [82]. In his protocol, a mixture of benzyl *N*-(fluorosulfonyl)carbamate and its lithium salt served as the external nitrogen nucleophile and Selectfluor as the co-oxidant. *N*-allyl benzamide derivatives as well as *N*-allyl aliphatic amides were suitable substrates for the process. These conditions promote the formation of oxazolines **78** in good yields and enatioselectivities of 48–77%.

**Scheme 40:**
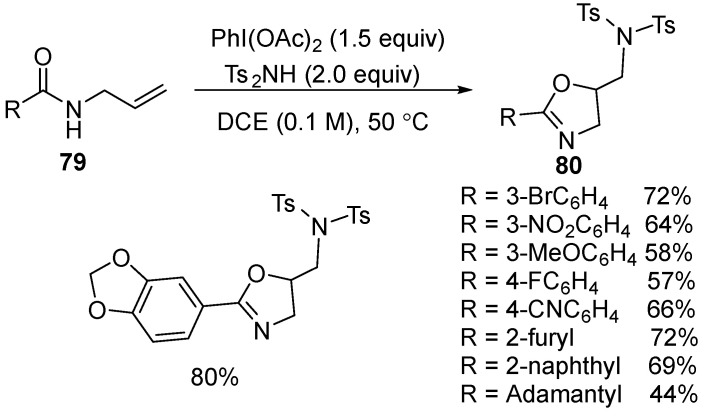
Synthesis of aminomethyloxazolines.

Another synthetic procedure for the preparation of aminomethyloxazolines from *N*-allyl amides **79** and bis(arylimides) as a nitrogen source mediated by (diacetoxyiodo)benzene has been reported (Scheme 40) [83]. Generated in situ, PhI(NTs_2_)_2_ or PhI(OAc)(NTs_2_) activated the olefin in intramolecular cyclization to furnish the 5-aminomethyloxazolines **80** in moderate to good yields.

**Scheme 41:**
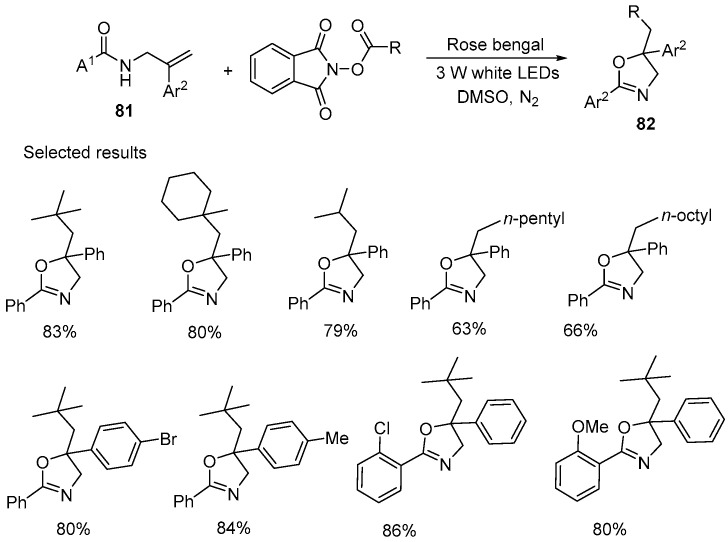
Photoinduced radical synthesis of 5-alkyloxazolines.

A variety of 5-alkyloxazolines **82** were produced in the photoinduced radical cyclization of *N*-allyl amides **81** with *N*-hydroxyphthalimide ester (NHP) (Scheme 41) [84]. Rose Bengal proved to be the most effective photocatalyst in the process when irradiated with 3 W white LEDs. The yields received were in the range of 63–88%.

**Scheme 42:**
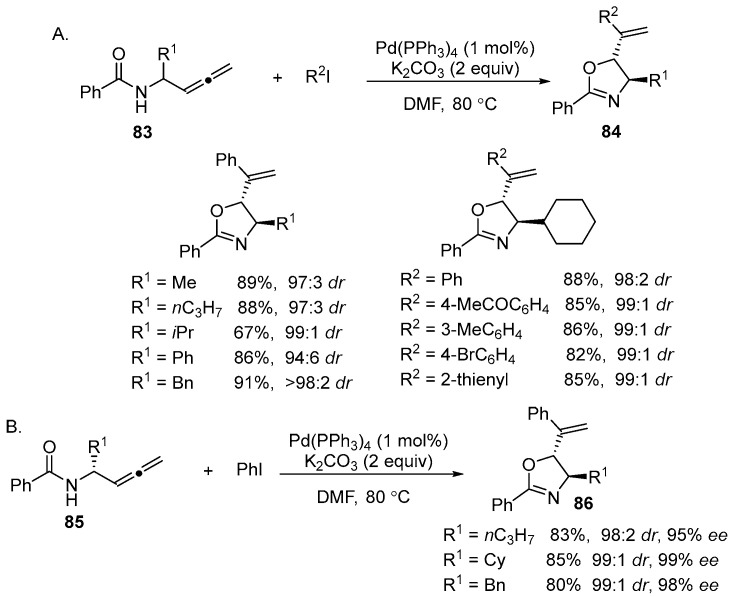
*Trans* diastereoselective synthesis of trisubstituted oxazolines: (**A**) from racemic *N*-(buta-2,3-dienyl)amides; (**B**) from enantiopure *N*-(buta-2,3-dienyl)amides.

The highly *trans* diastereoselective synthesis of trisubstituted oxazoline derivatives **84** through the palladium-catalyzed coupling–cyclization reaction of *N*-(buta-2,3-dienyl)amides **83** with aromatic iodides was elaborated by Wang (Scheme 42) [85]. Reactions catalyzed by Pd(PPh_3_)_4_ produced oxazolines **84** in excellent yields and diastereoselectivity. The application of optically active *N*-(buta-2,3-dienyl)amides **85** allowed the oxazolines **86** to be produced in high optical purity of 95–99%.

## 3. Synthesis of Oxazolines from *N*-Propargyl Amides

### 3.1. Gold-Catalyzed Electrophilic Intramolecular Cyclization of N-Propargyl Amides

**Scheme 43:**
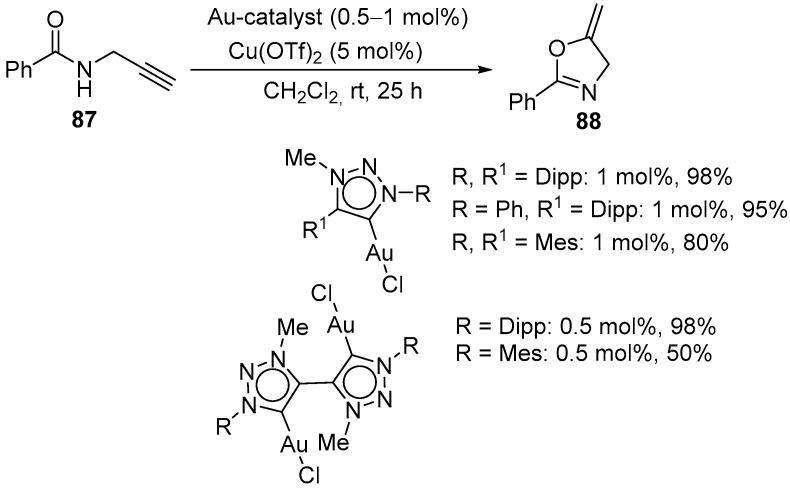
Cyclization of *N*-(2-propyn-1-yl)benzamide to 5-vinylidene-2-oxazoline catalyzed by Au(I)-1,2,3-triazol-5-ylidenes.

Gold(I) complexes are the most effective catalysts for electrophilic activation of a triple bond [86,87]. They have therefore been proven to be effective catalysts for the intramolecular cyclization of *N*-propargylic amides to oxazolines, while Au(III) complexes direct the cyclization towards oxazoles [88,89]. A plethora of work has recently been performed to understand the relationship between the structure of the gold catalyst and the mechanism of the cyclization of the *N*-propargylic amide [90,91,92,93,94]. For catalysis with gold(I) complexes, moisture- and light-sensitive silver salts are usually used as activators. Hettmanczyk replaced them with a more stable copper in the intramolecular cyclization of *N*-(2-propyn-1-yl)benzamide **87** to 5-vinylidene-2-oxazoline **88** catalyzed by Au(I)-1,2,3-triazol-5-ylidenes (Au(I)-MIC) mono- and digold complexes (Scheme 43) [95]. Depending on the structure of the MIC ligand and the catalyst loading, the oxazoline was formed with a 50–98% yield.

**Scheme 44:**
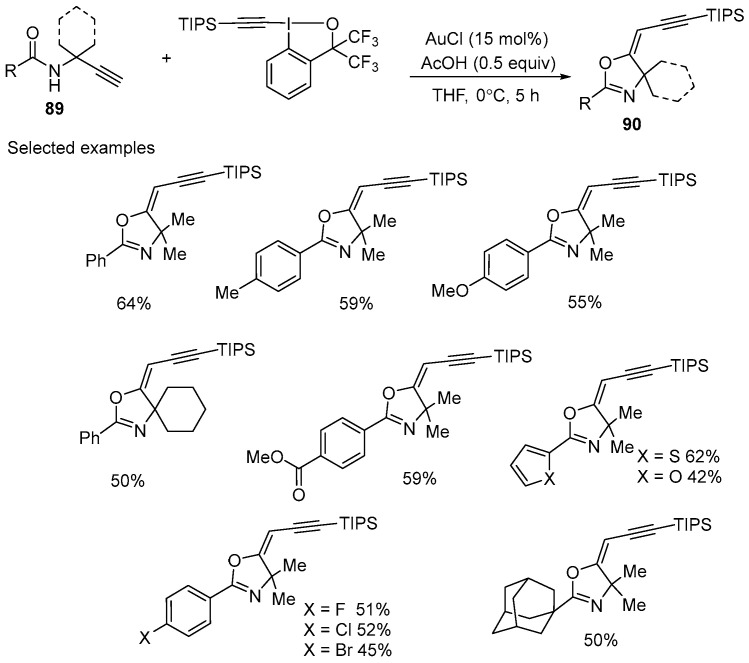
Cyclization–alkynylation of *N*-propargylic amides with benziodoxole reagent.

In 2019, a highly stereoselective synthesis of alkynyloxazolines **90** via a gold-catalyzed domino cyclization–alkynylation cascade of *N*-propargylic amides **89** with a benziodoxole reagent was reported by Hashmi (Scheme 44) [96]. A wide range of substrates with a terminal triple bond can be used in this method. This methodology provides products of *E*-geometry with excellent stereoselectivity and good yields.

**Scheme 45:**
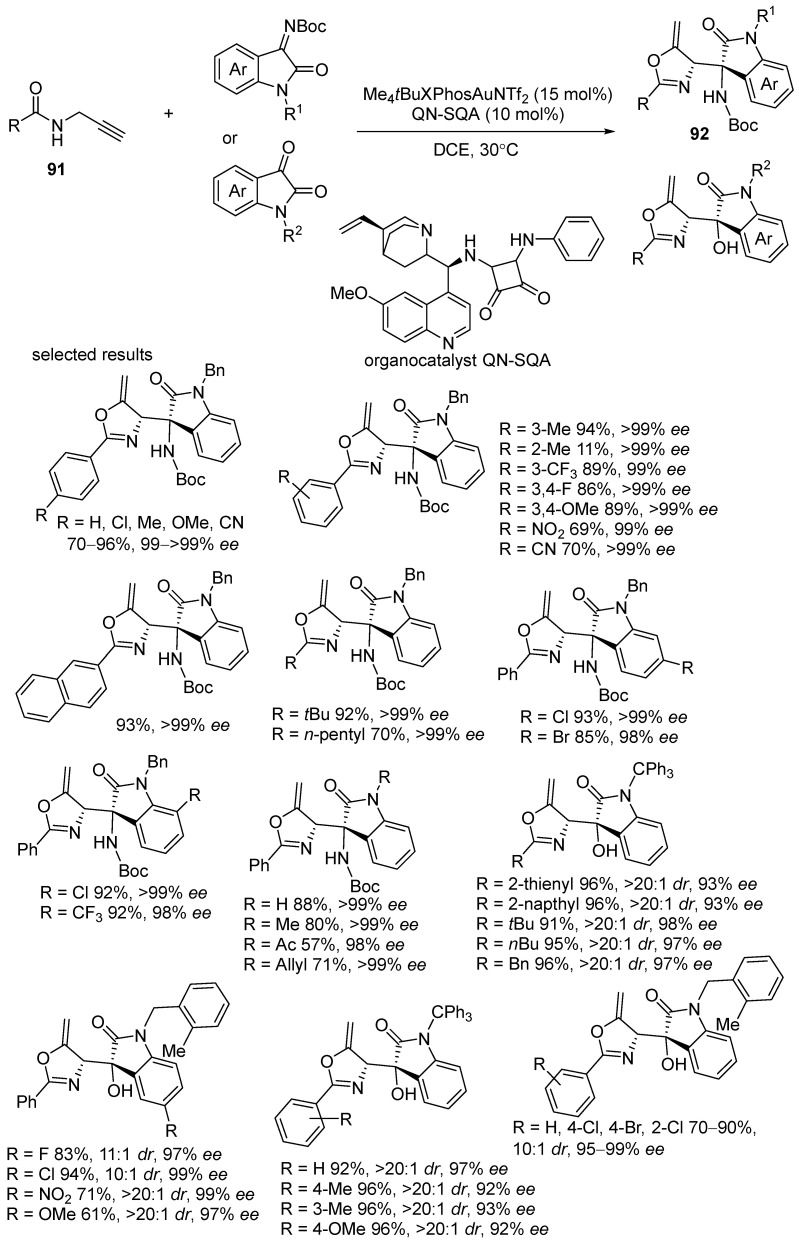
Enantioselective synthesis of 2,5-disubsituted alkylideneoxazolines.

A highly stereoselective cyclization of *N*-propargyl amides **91** and isatine or isatine ketimines enabled by gold and organo cooperative catalysis provided quick and easy access to chiral 2,5-disubsituted alkylideneoxazolines **92** (Scheme 45) [97].

**Scheme 46:**
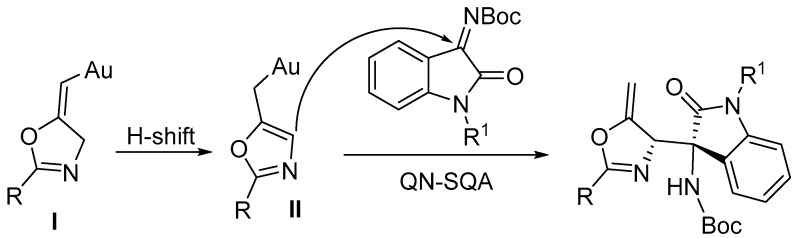
Mechanism of enantioselective cyclization of *N*-propargyl amides to 2,5-disubsituted alkylideneoxazolines.

A combination of Me_4_tBuXPhosAuNTf_2_ and quinine-derived squaramid (QN-SQA) organocatalyst is the most active catalyst system for cyclization. A plethora of structurally diverse amides **93** were cyclized to the respective optically pure oxazolines **94** in good to excellent yields. The mechanism of the reaction involves the formation of the (*E*)-vinylgold **I** intermediate which undergoes the H-shift process to form species **II**. Then the stereoselective asymmetric electrophilic allylation process through a formal hetero-ene reaction takes place with the assistance of the chiral organocatalyst (Scheme 46).

**Scheme 47:**
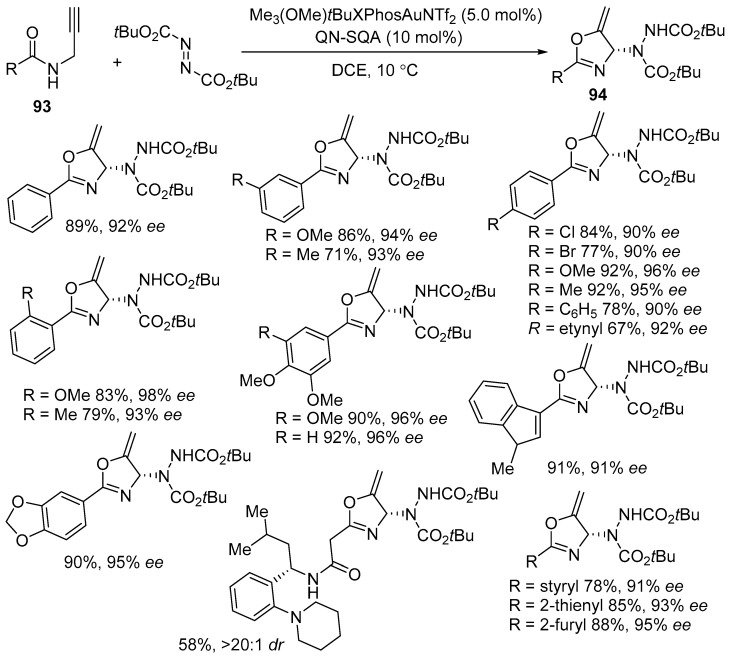
Enantioselective synthesis of chiral alkylideneoxazolines with a nitrogen-containing tertiary carbon stereocenter.

A similar catalytic system was involved in the enantioselective synthesis of chiral alkylideneoxazolines **94** with a nitrogen-containing tertiary carbon stereocenter [98]. The synthesis was performed upon the gold- and organo-catalyzed intramolecular aminocyclization of *N*-propargyl amides using azodicarboxylates as the *N*-source in the presence of bifunctional quinine-derived squaramid as oranocatalyst. A broad range of *N*-propargylic amides **93** delivered the products in good to excellent yields with 81%−98% *ee* (Scheme 47).

### 3.2. Silver- and Copper-Catalyzed Electrophilic Intramolecular Cyclization of N-Propargyl Amides

**Scheme 48:**
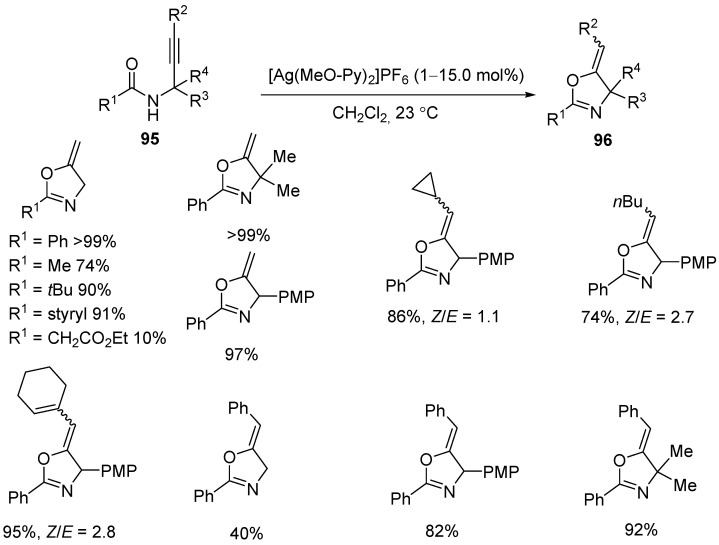
Catalytic cyclization of *N*-propargyl amides in the presence of silver catalyst.

Silver(I) complexes constitute another group of active catalysts for the 5-*exo*-*dig* cyclization of *N*-propargylic amides to oxazolines. Wong et al. synthesized a series of Ag(I)-(bis-pyridyl) complexes and applied them to the intramolecular cyclization of *N*-propargyl amides. The Ag-catalyzed cyclization of internal and terminal *N*-propargylic amides **95,** conducted under mild conditions in the presence of these electron-rich pyridyl ligands, yielded 5-alkylidene oxazolines **96** as the sole product in moderate to good yields (Scheme 48) [99]. Aromatic and aliphatic amides were good substrates under these conditions. Substrates containing ester, vinyl, and ethoxy substituents at R^1^ were ineffective in the reaction. Alkyl substituted at R^2^ amides gave the products as mixtures of *Z/E*-isomers, while for substrates with the phenyl ring, only the *Z*-isomer was obtained.

**Scheme 49:**
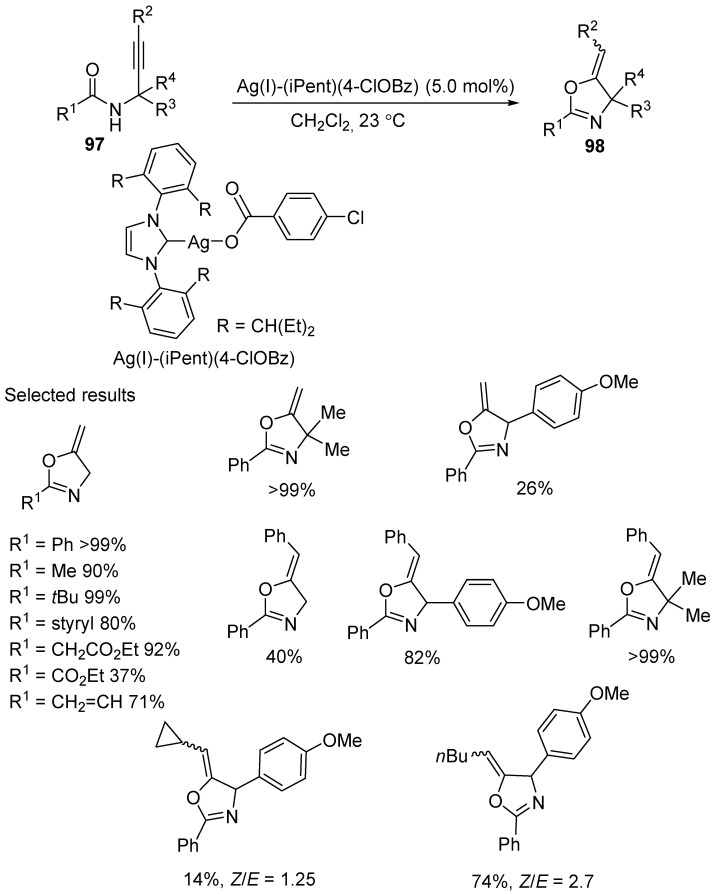
Silver-catalyzed cyclization of *N*-propargyl amides.

**Scheme 50:**
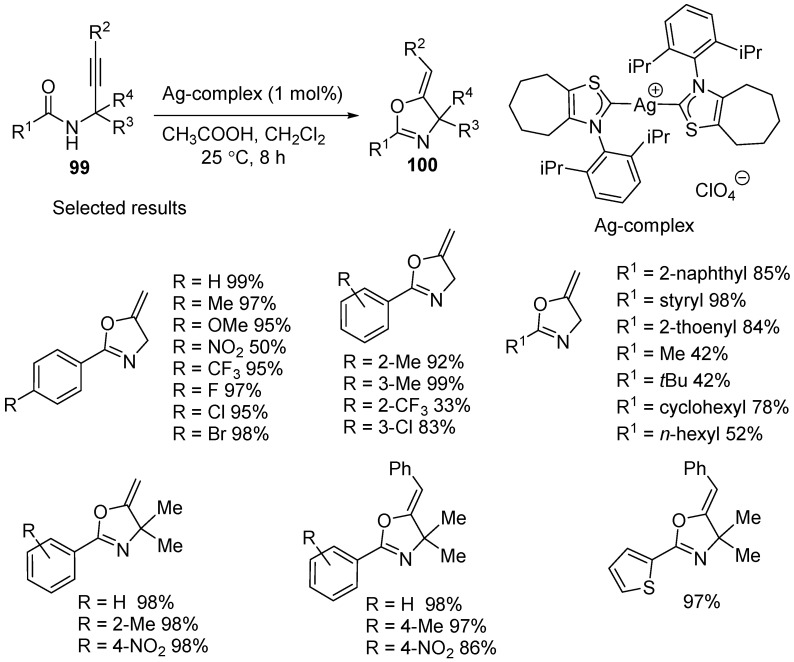
Ag(I)–thiazol-2-ylidene complex-catalyzed synthesis of methylideneoxazolines.

Wong also studied the structural and electronic features of the series of Ag(I)–(NHC)(O_2_CR) complexes [100]. In these complexes, the NHC ligand is responsible for the electronic and steric properties, while the carboxyl ligand is responsible for their stability. Ag(I)-(IPent) (4-ClOBz) was the most active in the intramolecular cyclization of nonterminal and terminal *N*-propargylic amides **97** (Scheme 49). Substrates containing the electron-withdrawing R^1^ (OEt, CO_2_Et) group underwent cyclization in the reaction catalyzed by the Ag-NHC catalyst in contrast to the reaction catalyzed by the Ag-(bispyridyl) catalyst. The two catalytic systems, Ag(I)-(bispyridyl) and Ag(I)-(NHC)(O_2_CR), have highly complementary reaction scopes. Cyclization catalyzed by the Ag(I)–thiazol-2-ylidene complex produced 5-methylidene oxazolines **100** in excellent yields at room temperature (Scheme 50) [101]. These catalytic conditions were active for amides **99** with both terminal and internal triple bonds. The mild reaction conditions were applied to the late-stage functionalization of some biologically active compounds possessing sensitive functional groups such as halides, cyano, nitro, sulfonamide, aryl ethers, and S-heterocycles.

**Scheme 51:**
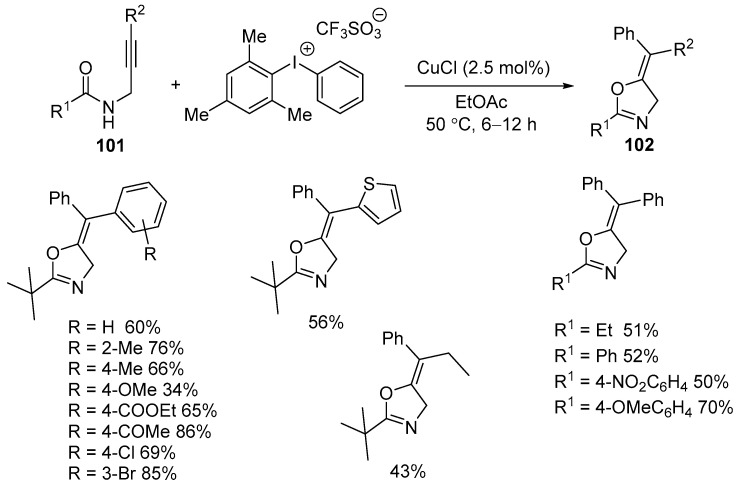
Synthesis of 5-alkylideneoxazolines with tetrasubstituted exo double bond.

5-Alkylideneoxazoline derivatives **102** with tetrasubstituted exo double bond were obtained in good yields in the copper-catalyzed reaction of *N*-propargylic amides **101** and diaryliodonium salts (Scheme 51) [102]. This transformation was successfully achieved using aromatic and aliphatic amides with a substituent in the distal position of the triple bond. Various substituents were tolerated in the *para*- and *meta*-positions of the phenyl group of the iodonium salt under reaction conditions, regardless of their electronic nature. After activation of the triple bond by the Cu(III) catalyst, two possible mechanistic pathways of cyclization can occur, according to the authors. One involves a nucleophilic attack of the carbonyl oxygen atom on a copper(III)−acetylene π complex; the other attack on the carbocation formed by the opening of the copper(III)−acetylene π complex. Two routes of the reductive elimination step are also presented.

### 3.3. Transition Metal-Free Electrophilic Intramolecular Cyclization of N-Propargyl Amides

**Scheme 52:**
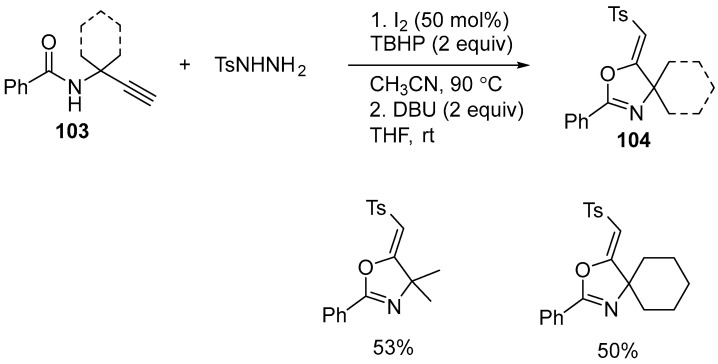
Iodine-promoted regioselective cyclization of *N*-propargylic amides with sulfonyl hydrazides.

Electrophilic intramolecular cyclization of *N*-propargylic amides can be carried out under transition-metal catalyst-free conditions with the use of Lewis or Brønsted acid or another activator. For example, synthesis of oxazolines **104** substituted with a vinyl sulfone moiety at C5 was accomplished by the iodine-promoted regioselective cyclization of *N*-propargylic amides **103** with sulfonyl hydrazides (Scheme 52) [72]. Only amides possessing a quaternary propargylic carbon atom can provide oxazolines in this process.

**Scheme 53:**
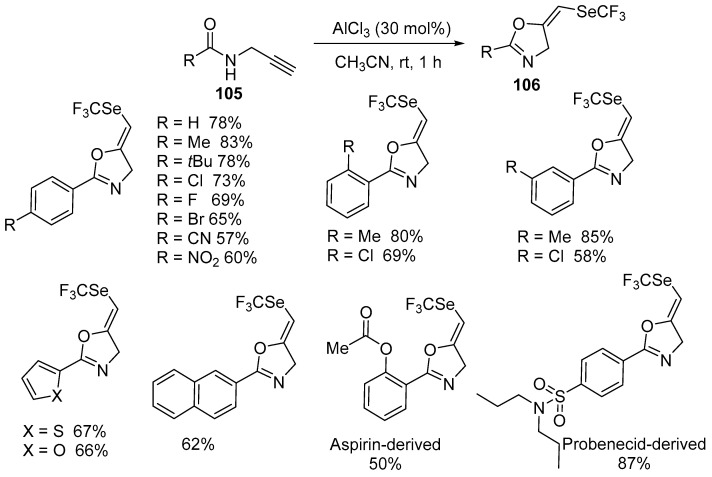
AlCl_3_-catalyzed synthesis of SeCF_3_-substituted oxazolines.

The electrophilic trifluoromethylselenolation cyclization of *N*-propargylic amides **105** induced by *N*-trifluoromethylselenophthalimide under Lewis acid catalysis has been disclosed very recently (Scheme 53) [103].

**Scheme 54:**
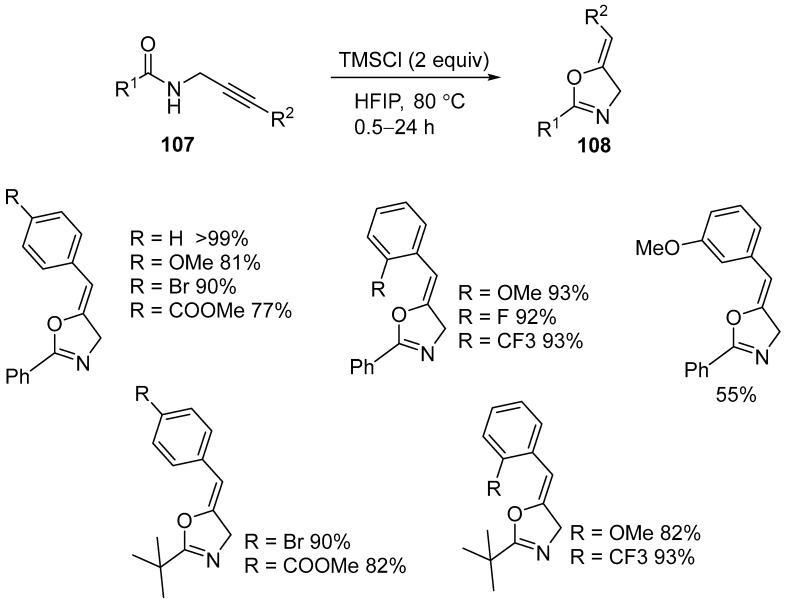
Synthesis of (*Z*)-configured alkylideneoxazolines.

Substrates with terminal alkyne underwent the 5-*exo-dig* cyclization that yielded SeCF_3_-substituted oxazolines **106** with moderate to good yield. The 6-*exo-dig* cyclization followed by isomerization to oxazoles occurred while amides with internal alkyne were subjected to the reaction (see Scheme 2). The potential usefulness of this method has been demonstrated by successfully conducting experiments on a gram scale and late-stage functionalization of various biologically significant derivatives. Jankowski developed an efficient transition-metal-free protocol for the stereoselective cyclization of propargyl amides **107** with internal triple bonds. According to the protocol, the synthesis of 2-oxazolines **108** was performed in the presence of TMSCl as a source of anhydrous HCl in hexafluoroisopropanol (HFIP) (Scheme 54) [104].

The reaction is highly stereoselective, providing oxazolines **108** that exhibit the (*Z*)-configuration of the exocyclic double bond. The cyclization rate is strongly dependent on the electronic character of the substituent in the benzene ring at the distal end of the triple bond. For the benzamide derivatives, the *orto*- and *para*-methoxy-substituted amide afforded oxazoline in the yield of 93% and 81% after 30 min at room temperature. The methoxy group in the *meta* position decreased the yield to 55%. Electron-withdrawing trifluoromethyl and ester groups decreased the reaction rate, so the cyclization took 24 h at 80 °C to produce oxazolines with a 93% and 71% yield, respectively. Very good reactivity was observed in the reactions of halogenated amides, which provided an excellent yield of oxazolines after 1.5 h at 80 °C. Pivalamide derivatives also exhibited good reactivity in cyclization. When propargylic amides bearing nitrile or nitro groups were applied to the conditions, a mixture of oxazolines and oxazoles was obtained.

## 4. Conclusions

This short review summarizes synthetic procedures for the preparation of oxazolines that utilize electrophilic intramolecular oxidative cyclization of *N*-allyl and *N*-propargyl amides. Both substrates give the 5-substituted derivatives of oxazoline. Intramolecular oxidative cyclization of *N*-allyl amides provides access to a variety of oxazolines substituted at the 5 position of the ring, with functional groups that are important in light of their biological properties. Furthermore, the cyclization performed under enantioselective control gives the opportunity to obtain enantioenriched or optically pure derivatives. On the other hand, methylidene substituted at C-5 oxazolines are formed in the intramolecular oxidative cyclization of *N*-propargyl amides. The direction of the process towards the *E* or *Z* diastereoizomer is possible by applying the appropriate reaction conditions. It is worth noting that, under suitable conditions, the cyclization of *N*-propargyl amides can become a tool for enantioselective formation of a stereogenic centre at the C-4 position of oxazoline.

Undoubtedly, a major limitation of syntheses based on the intramolecular cyclization of *N*-unsaturated amides is the synthesis of appropriately substituted starting *N*-allyl and *N*-propargyl amides. Regiochemistry and the enantioselective outcome of the cyclization depends on the amide’s substitution pattern. In recent years, there has been growing interest in electrochemistry, particularly in electrochemical oxidation as an important green tool for organic synthesis. Therefore, the electrochemical cyclization of *N*-allyl amides may constitute a convenient, environmentally friendly method for the synthesis of oxazoline derivatives in the future. This green method does not require the application of oxidants, transition metal catalysts, or elevated temperatures. However, this methodology can be associated with difficulties in scaling it up to a gram-scale reaction. In the future, *N*-propargyl amide cyclization that does not require expensive gold or silver catalysts will likely attract more interest. Thus, Lewis or Brønsted acid-catalyzed reactions, hypervalent iodine-catalyzed reactions, and other promoters are promising tools for the synthesis of oxazolines from *N*-propargyl amides.

We assume that electrophilic intramolecular oxidative cyclization of *N*-allyl and *N*-propargyl amides will continue the attraction as a synthetic method of oxazolines. The scope of this synthetic method can be expanded by introducing boron- or phosphorus-centred electrophiles. This could lead to the formation of oxazolines with boron- or phosphorus-containing groups. Since boron and phosphorus organic compounds have various therapeutic applications, these oxazoline derivatives may exhibit interesting biological properties. The regioselectivity of the intramolecular cyclization of *N*-allyl and *N*-propargyl amides is one of the most crucial aspects when it comes to synthesis. Machine learning (ML), a tool that has proven to be useful for predicting the outcome of organic reactions, can be helpful for predicting the regioselectivity of cyclization [105]. Therefore, the future challenge is to develop an LM algorithm that can predict the regioselectivity of the intramolecular cyclization of *N*-allyl and *N*-propargyl amides.

## Figures and Tables

**Table 1:** Comparison of Toste and Hirokawa methods of enantioselective synthesis of bromodifluoromethyloxazoline derivatives.

	Toste Method	Hirokawa Method
Brominating reagent	[(DAB)_2_Br](BF_4_)_3_	DBDMH
Brominating reagent loading	1.2 equiv.	1.5 equiv.
Phase-transfer catalyst	(*R*)-TRIP	POHOP
Phase-transfer cat. loading	10 mol%	10 mol%
Solvent	Toluene	Dichloromethane
Temperature	rt	−78 °C
Base	K_3_PO_4_	-
Yield	33–79%	44–98%
*ee*	60–92%	92–99%

**Table 2:** Comparison of organocatalytic asymmetric chloro- and bromocyclization of allylic amides to chiral 5-chloromethyl- and 5-bromomethyloxazolines.

	Rassias Chlorocyclization	Hamashima Bromocyclization
Halogenating reagent	NCS	NBS
Halogenating reagent loading	1.3 equiv.	1.2 equiv.
Organocatalyst	(*S*)-(+)-DTBM-SEGPHOS	(S)-DTBM-BINAP
Organocatalyst loading	10 mol%	10 mol%
Solvent	CpMe	Dichloromethane
Temperature	5 °C	−78 °C
Time	16 or 18 h	12–24 h
Yield	40–79%	40–99%
*ee*	20–97%	39–99%

**Table 3:** Methods of trifluoromethylation of *N*-allyl amides.

Entry	Substrate	CF_3_ Source	Conditions	Product	Yield
1	**31**	CF_3_SO_2_Na	PhI(OAc)_2_, DCE 40 or 90 °C	**32**	48–80%
2	**33**	CF_3_SO_2_Na	C(+)‖Pt(−) K_2_CO_3_ (2 equiv)) MeCN:H_2_O = 4.5:1 8 mA, rt, 4 h	**34**	55–82%
3	**35**	CF_3_SO_2_Na/ CF_2_HSO_2_Na	CF(+)‖Ni(−) Bu_4_NPF_6_, HFIP 5 mA, 60 °C, 4–6 h	**36**	33–82%
4	**37**	Umemoto reagent	[Ru(bpy)_3_](PF_6_)_2_ (0.5 mol%) 2,6-lutidine (1.1 equiv) Acetone or DCM 425 nm blue LEDs −78 °C, 3 h	**38**	46–86%
5	**39**	Togni reagent	KI (2 equiv) Dioxane, 80 °C, 6 h	**40**	39–86%
6	**41**	Togni reagent	Co(salen) (7 mol%) MeCN, 80 °C, 3 h	**42**	57–97%

**Table 4:** Summary of methods of the synthesis of sulfonyl functionalized oxazolines.

Entry	Substrate	Sulfonyl Source	Conditions	Product	Yield
1	**43**	R^1^SO_2_Na	Ag(OAc)_2_ (2 equiv) MeCN, 80 °C	**44**	70–91%
2	**45**	Ar^1^SO_2_NHNH_2_	C(+)‖Pt(−) KMe_4_NBF_4_ (0.17 M)) MeCN (3 mL) 15 mA, rt, 2 h	**46**	39–94%
3	**47**	DABCO·(SO_2_)_2_	Ar^1^N_2_BF_4_ *fac*-Ir(ppy)_3_ (2 mol%) 6 W blue LEDs (445–450 nm) Dioxane/MeCN rt, 12 h	**48**	33–82%

**Table 5:** Summary of methods of the synthesis of sulfenyl-functionalized oxazolines.

Entry	Substrate	Sulfenyl Source	Conditions	Product	Yield
1	**49**	ArS-succinimide	BF_3_·OEt_2_ DCE, 105 °C 24 or 36 h	**50**	36–92%
2	**51**	ArS-succinimide	TBAC (20 mol%M) CSA (20 mol%) DMF, 40 °C, 24 h	**52**	62–97%
3	**53**	ArSSAr	0.76 F/mol Bu_4_NBF_4_/DCM −78 °C, 30 min.	**54**	72–88%
4	**55**	4-methylbenzenethiol	C(+)‖Pt(−) Bu_4_NBF_4_/MeCN 20 mA, 40 °C, 5 h	**56**	66–82%
5	**57**	RSO_2_NHNH_2_	I_2_ (1 equiv) Toluene 120 °C, 5–12 h	**58**	40–65%
6	**59**	*N*-trifluoromethylthiosaccharin	*fac*-Ir(ppy)_3_ (2 mol %) 6 W blue LED MeCN	**60**	64–88%

**Table 6:** Summary of methods of the synthesis of selenyl-functionalized oxazolines.

Entry	Substrate	Selenyl Source	Conditions	Product	Yield
1	**61**	ArSeSeAr	Undivided cell C(+)‖Pt(−) LiClO_4_,(0.1 mol) 15 mA, MeCN, rt	**62**	63–91%
2	**63**	ArSeSeAr	Undivided flaw cell Graphite electrodes LiClO_4_,(0.02 M) MeCN:TFE 9:1	**64**	50–90%
3	**65**	PhSeSePh	PhIO (1 equiv) DCM, rt	**65**	71–90%

## Data Availability

No new data were created or analyzed in this study. Data sharing is not applicable to this article.
